# A Study of Differential Topology on the Magnetically Induced Isotropically Averaged Lorentz Force Density of a Few Simple Molecules

**DOI:** 10.3390/molecules29184502

**Published:** 2024-09-23

**Authors:** Michele Orza, Francesco F. Summa, Riccardo Zanasi, Guglielmo Monaco

**Affiliations:** Dipartimento di Chimica e Biologia “Adolfo Zambelli”, University of Salerno, Via G. Paolo II, 123, 84184 Fisciano, SA, Italy; michele.orza@unibo.it (M.O.); fsumma@unisa.it (F.F.S.); rzanasi@unisa.it (R.Z.)

**Keywords:** Lorentz force density, quantum chemical topology, topological index, Poincaré–Hopf theorem, vector field

## Abstract

Quantum chemical topology addresses the study of the chemical structure by applying the tools of differential topology to scalar and vector fields obtained by quantum mechanics. Here, the magnetically induced isotropically averaged Lorentz force density was computed and topologically analyzed for 11 small molecules. Critical points (attractors, repellers, and saddles) were determined and trajectories connecting the attractors computed. It is shown that kinds and numbers of the critical points are to some extent transferable in similar molecules. CC bonds of different orders are endowed with critical points of different kinds close to their center. The sum of topological indices of the isolated critical points is influenced by the presence of repellers on the outer part of the molecules.

## 1. Introduction

Quantum chemical topology (QCT) is the field of research intending to study, via differential topology, scalar and vector fields, which are useful to characterize molecules [1,2,3]. It has its origin in the work of Bader, who first showed that basic features of a chemical structure, i.e., atoms, bonds, faces, and cages, can be associated to the occurrence of critical points (CPs) of the gradient of the electron density, i.e., the points where ∇ρ=0 [4,5]. The location of the maxima of the density turned out to be unexpected (non-nuclear maxima) only in few special though interesting cases [6,7,8]. On the other hand, the possibility to identify the most relevant atomic interactions, the “chemical bonds”, via a topological analysis, i.e., by assessing whether the maxima of the electron density of two atoms are connected by a trajectory passing through the so-called Bond Critical Point (BCP), has inspired an amazingly long list of contributions in the literature. The case of the H-H bonding, to cite just one of the many discussed systems, has been thoroughly discussed [9,10,11,12,13,14,15,16,17]. It has been shown that the onset of energy differences historically attributed to H-H steric clashes are well understood in terms of destabilizing interactions of C atoms carrying those H atoms, which can well have a slight stabilizing interaction. The case well testifies how much the chemical community is reluctant to consider any alternative paradigm on bonding. In the spirit of the original Atoms In Molecules (AIM) theory [4], the topological analysis of ∇ρ is sufficient to extract a *molecular graph*, capturing the main elements of the molecule: the atoms and the bond paths linking a subset of atom pairs. Although the choice of the density gradient as the field to unravel the chemical structure finds its physical justification in its homeomorphism with the virial field, i.e., the electronic potential energy density of an electron [18], and in the identification of density-driven bond paths as privileged exchange channels [19,20], different fields have also been used to that end [21]. We recently studied the magnetically induced isotropically averaged Lorentz force density $\langle F\rangle$ [22], which offers a promising avenue to reveal the chemical electronic structure starting from the magnetic response.

In the present paper, we give a thorough discussion of the topology of $\langle F\rangle$ in a few simple molecules built from atoms of the first and second period, aiming at showing some of their features in the realm of QCT. The attractors of $\langle F\rangle$ occur in correspondence of both atoms and bonds, and thus their connection, as captured by trajectories passing through the saddles equivalent to the BCPs, can be expected to be more complicated than that of ∇ρ, likely with some similarity to that obtained from the Laplacian of the density. The latter has been used to introduce the L−graphs [23], and, by analogy, we will here introduce $\langle F\rangle -$graphs. The analysis will be enriched with a discussion on the count of critical points, whose topological indices should be bound by relations of the kind of the Poincaré–Hopf theorem.

We will first present in Section 2 a systematic report of the topological features of $\langle F\rangle$ computed in prototypes of acyclic molecules (lithium hydride, methane, ethane, ethylene, acetylene, propyne, and 3-borapropyne), aromatic, antiaromatic and non-aromatic molecules (benzene, planarized cyclooctateraene, and borazine), and a cage molecule (cubane), and we will then discuss and resume the main finding in Section 3. Although, the theory on the Lorentz force density and on topology can be found in dedicated papers and textbooks, a short description of them will be found in Section 4 for the benefit on non-specialized readers. Then, after some computational details, we will arrive at our conclusions in Section 5.

## 2. Results

We are going now to report the location and number of the critical points (CPs), i.e., the points in space where $\langle F\rangle =0$, found for the molecules chosen. A summary of the results is given in Table 1. Lists of the location and eigenvalues of the Jacobian at the CPs are given in the Supporting Information (Table S3).

### 2.1. Lithium Hydride

**Figure 1:**
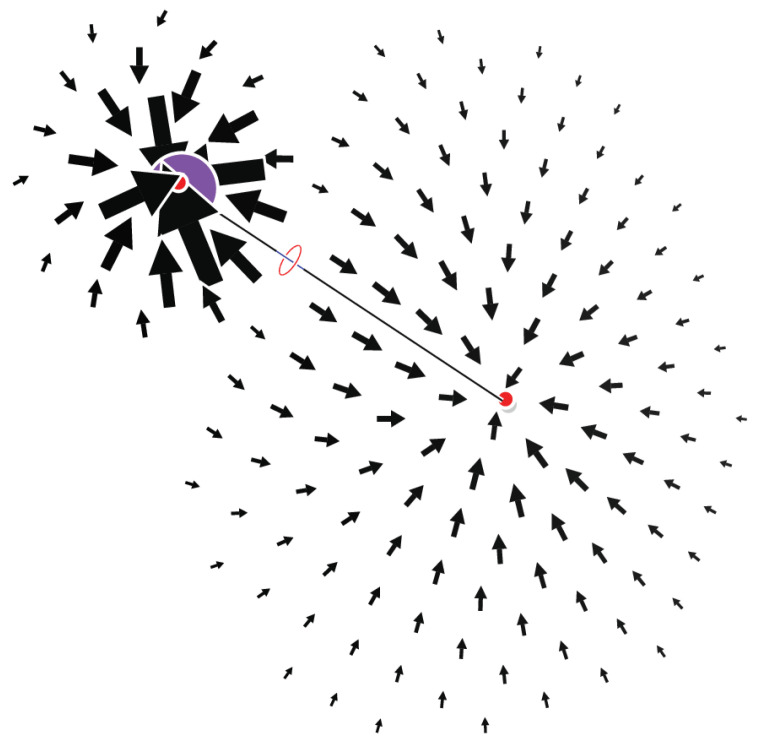
The field $\langle F\rangle$ plotted on a plane containing the LiH bond. The critical points are displayed according to the convention given in the Computational Methods section.

As a first example, we show the $\langle F\rangle$ field for LiH (Figure 1). In this simple molecule, the field has two attractors separated by a (3,−1) CP. The field is inward-oriented in all places (on a boundary containing all isolated CPs) and the sum of indices is consistently equal to −1.

### 2.2. Methane

**Figure 2:**
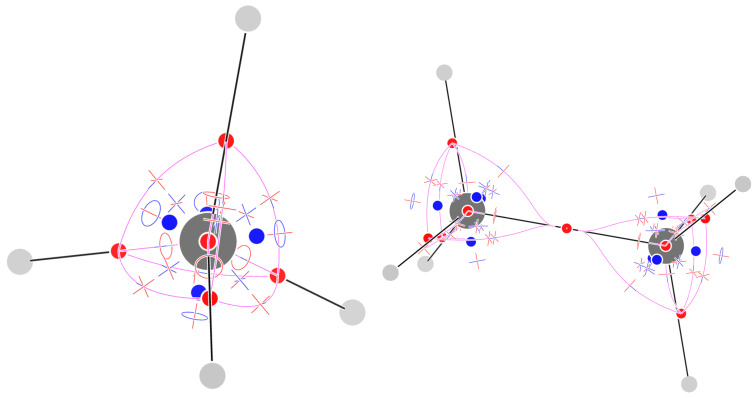
Critical points of $\langle F\rangle$ and trajectories defining the $\langle F\rangle -$graph for methane (**left**) and staggered ethane (**right**). Please see the Computational Methods section for graphical conventions.

Figure 2 shows the 29 critical points of $\langle F\rangle$ for methane (their list is given in the Supporting Information). There are five (3,−3) attractors, one on the C atom, and four on the C–H σ bonds. If connected by trajectories passing through the (3,−1) CPs, they form a tetrahedral shaped complete graph $K_{5}$: every attractor is bound to any other one, and indeed the number of (3,−1) CPs is n2=52=10. The number of topologically triangular faces is also n1=53=10 and a (3,+1) face critical point can be associated to each of them. Eventually, the $n_{0}=4$(3,+3) CPs can be associated to tetrahedral cages formed by three H atoms and the central C atom. The sum of indices is −1 as expected for an inward-oriented vector field, with a single repeller at infinity.

The complete graph $K_{5}$ is one of the two basic gt-nonplanar graphs, i.e., those that cannot be drawn on a sheet of paper without crossing of edges. gt-nonplanar graphs are unusual topologies in organic chemistry [24,25], when one considers standard bonding schemes, often compatible with the molecular graphs obtained from ∇ρ. However, we see that even the simplest organic molecule, namely, methane, shows a gt-nonplanar graph, when studied with the $\langle F\rangle$ field.

### 2.3. Ethane

Figure 2 shows the 57 critical points of $\langle F\rangle$ and the trajectories defining the $\langle F\rangle -$graph for staggered ethane. All topological features are preserved in eclipsed ethane (see the Supporting Information, Figure S1). The number of CPs is just one less than the sum of the CPs of two methane molecules: indeed one of the (3,−3) CPs is shared between the two methyl moieties. The methyl unit has, thus, a conservative pattern of CPs, much as reported for the Laplacian of the density [23]. The sum of indices equals −1, as expected for an inward-oriented vector field.

### 2.4. Ethylene

**Figure 3:**
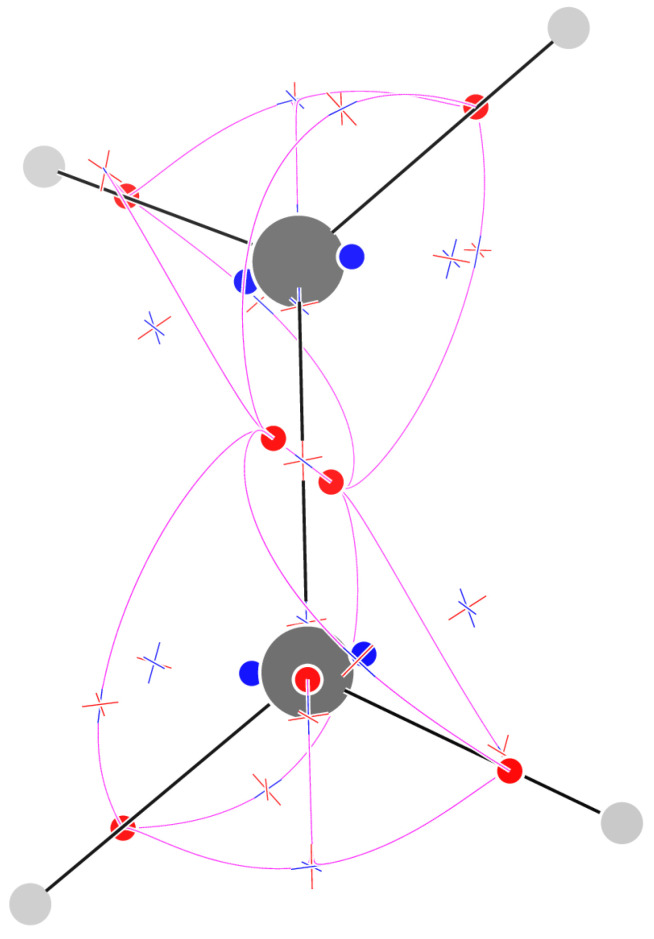
Critical points of $\langle F\rangle$ and trajectories defining the $\langle F\rangle -$graph for ethylene.

The $\langle F\rangle$ field for ethylene has 31 isolated CPs (Figure 3). Eight CPs are attractors of $\langle F\rangle$: two on the C atoms, four on the C–H σ bonds, and a pair above and below the molecular plane in correspondence with the double bond. As a consequence, in the middle of the C–C bond, rather than finding an attractor, we find a (3,−1) CP. In addition to these CPs, on each methylene moiety, we find five (3,−1), four (3,+1) and two (3,+3) CPs. The (3,−1) CPs are half of those found in the methyl moiety in ethane: The CH attractors are linked to the π-bond attractors from above and below (four trajectories for each methylene); the (3,−1) CP close to the C attractor, in the HCH region, has trajectories going on either of the close CH attractors, separated by a single trajectory that goes directly in the (3,+1) CP. The (3,+3) CPs appear between two topological triangles formed by the two π−bond attractors and either the CH-bond attractor or a (3,+1) CP located close to the C atom. The sum of indices is −1 as expected for an inward-oriented vector field.

### 2.5. Acetylene

**Figure 4:**
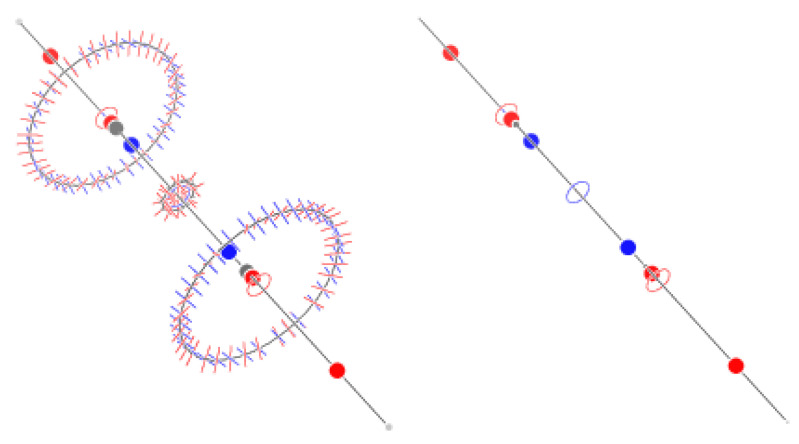
Critical points of $\langle F\rangle$ for acetylene. On the right, only the 9 critical points along the molecular axis are shown, and the gray spheres centered on C and H nuclei have been scaled down.

The $\langle F\rangle$ field of acetylene is not formed by isolated CPs only, but three circles of critical points appear: a circle of (2,−2) CPs in the middle of the molecule and two circles of (2,0) CPs close to the location of C atoms (Figure 4). Along the molecular axis, nine CPs are found: four (3,−3) CPs located on C atoms and on C–H bonds, a (3,+1) CP in the middle of the C–C bond, two (3,+3) CPs between the middle of the C–C bond and the C atoms, and two (3,−1) CPs between the C atoms and the attractors on C–H bonds. The latter (3,−1) CPs are only 0.097 au away from the (3,−3) CPs located on C atoms. Disregarding the continuous circles of CPs, the sum of indices is −1 as in all previous molecules. It can be noted that, as far as the change in the vector field is considered only along the molecular axis, the sequence of the three CPs (3,+3)−(3,+1)−(3,+3) is equivalent to a sequence (3,−1)−(3,−3)−(3,−1), which could have been expected for a σ bond.

### 2.6. Propyne

**Figure 5:**
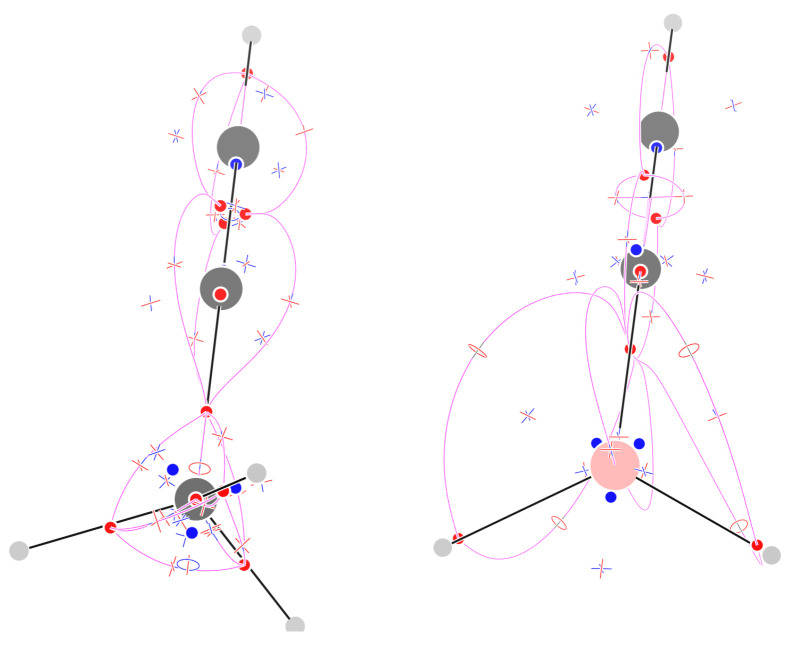
Critical points of $\langle F\rangle$ and trajectories defining the $\langle F\rangle -$graph for propyne (**left**) and 3-borapropyne (**right**).

The $\langle F\rangle$ field of propyne has 55 CPs (Figure 5). The insertion of the methyl has perturbed each of the three circles of rank-2 CPs of acetylene, producing two sets of three alternating CPs. In the case of the central circle of (2,−2) CPs, three (3,−3) points are generated alternating with (3,−1) CPs. In the case of the C-centered circles of (2,0) CPs, three (3,−1) CPs are formed alternating with three (3,+1) CPs. The sum of indices along each circle is zero. Excluding these 3×6=18 CPs, we have 37 CPs, just one less than the sum of the isolated CPs of acetylene and methyl: 9 + 29 = 38. Indeed, the location and kind of CPs are very similar to those of the smaller molecules, and the (3,−3) on the C-Me bond is shared by the two moieties.

### 2.7. 3-Borapropyne

The $\langle F\rangle$ field of 3-borapropyne has 43 isolated CPs (Figure 5). Seven out of the nine (3,−3) CPs occur close to atoms or to the centers of σ bonds. Two further attractors occur in the plane bisecting the HBH angle, in place of the circle of (2,−2) CPs found in acetylene. The (3,+3) repellers occur three around the B atom, two around the central C atom, and one close to the peripheral C atoms. Of the 15 (3,−1) CPs, 12 are out-of-plane (the 4 connecting the attractors on BH bonds and the B atom has two complex eigenvalues with a negative real component) and the remaining 3 occur on the symmetry axis. No trajectory passing through a (3,−1) CP links the BH attractors directly. Of the 13 (3,+1) CPs, 7 can be associated to loops of alternating (3,−3) and (3,−1) CPs, 3 alternate with repellers close to the B atom, 2 alternate with repellers close to the central C atom, and 1 acts as a repeller of trajectories (not shown)  originating from the (3,+3) CP close to the B atom along the bisector of the HBH angle.

### 2.8. Benzene

**Figure 6:**
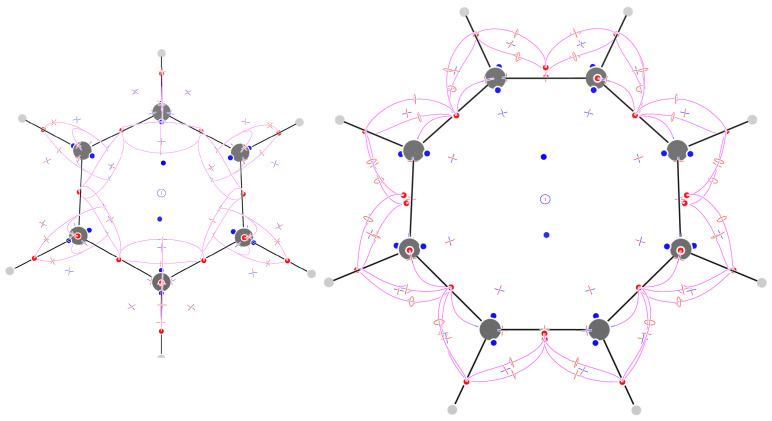
Critical points of $\langle F\rangle$ and trajectories defining the $\langle F\rangle -$graph for benzene (**left**) and cyclooctatetraene (**right**).

The $\langle F\rangle$ field of benzene has 99 CPs (Figure 6). The 18 (3,−3) CPs are located on C atoms, CH, and CC bonds. Of the 14 (3,+3) CPs, 12 are close to the C atoms, located towards the inside and the outside of the ring, and 2 are above and below the ring. The 36 (3,−1) CPs come from six sextets around the C atom. Each sextet, in turn, is composed of two triplets of CPs placed symmetrically above and below the ring. The three symmetry-unique (3,−1) CPs all occur in the plane of symmetry containing two opposing CH bonds, one very close to the C atom, one at some distance from the center of the CH bond, and one inside the ring, at a height of 0.952 bohr, close to the height traditionally chosen to display maps of the π current density [26,27]. Notably, along the CH bond, we do not find a (3,−1) CP, but the two attractors (the C attractor and the CH bond attractor) are separated by a (3,+3) CP. The four symmetry-unique CPs out of the thirty-one (3,+1) CPs occur as follows: one in the middle of the ring, one in the CH bond direction inside the ring, and two flanking the CH bond on either side.

**Figure 7:**
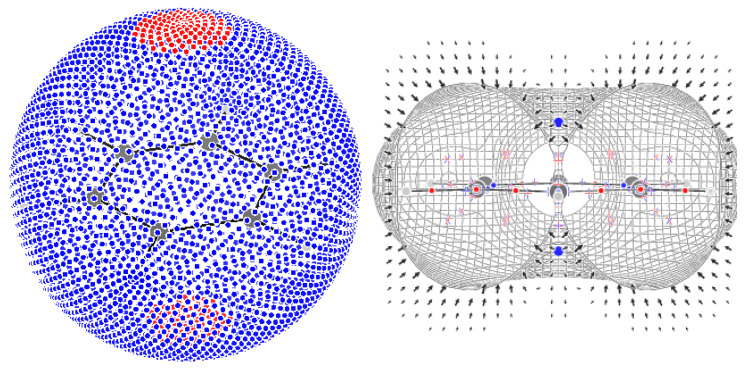
A sphere with a radius of 5 au with values of outward and inward flux of $\langle F\rangle$ represented as red and blue dots, respectively (**left**). A surface drawn at a value of $|\langle F\rangle |=0.001$ au encloses 97 out of the 99 CPs of benzene, leaving out only two (3,+3) CPs. The $\langle F\rangle$ field is inward-oriented in all places on the surface (**right**).

The sum of indices is +1. According to Leboeuf et al. [28], this value can be understood in terms of the presence of a repeller and two attractors at infinity: the map of the flux of $\langle F\rangle$ out of a spherical surface shows two zones of positive values inside a negative surface, which would be compatible with the presence of two asymptotic attractors and an asymptotic repeller (Figure 7). We find that a surface exists such that the field $\langle F\rangle$ is inward-oriented in all places. Such a surface excludes two repellers only; the sum of the index of the CPs inside the surface is then +1 − 2(+1) = −1, which is in agreement with the topological theory, and there is no need to invoke asymptotic CPs.

### 2.9. Cyclooctatetraene

The $\langle F\rangle$ field of planarized cyclooctatetraene has 123 CPs (Figure 6), with 28 (3,−3) CPs, occurring on atoms on CH and long CC bonds, and in pairs above and below the middle of short CC bonds. The trajectories following the eigenvector corresponding to the positive eigenvalue of the 44 (3,−1) CPs connect the C atom attractor with the CC long bond attractor, the two π-bond attractors, the CH-bond attractor with the adjacent CC bond, and π-bond attractors. The trajectories passing through the CH-bond attractor form topological triangles or squares each associated with a (3,+1) CP. Of the remaining 17 (3,+1) CPs, 16 occur in pairs close to the C atom, alternating with an equal number of (3,+3) CPs (much like the case of benzene, although with less symmetry), and 1 in the ring center separating 2 (3,+3) CPs located above and below the ring. The sum of indices is +1. This can be understood as in benzene, i.e., in terms of a surface enclosing all but two repellers, which generate two island-like regions of outward flux of $\langle F\rangle$ (see the Supporting Information, Figure S2).

### 2.10. Borazine

**Figure 8:**
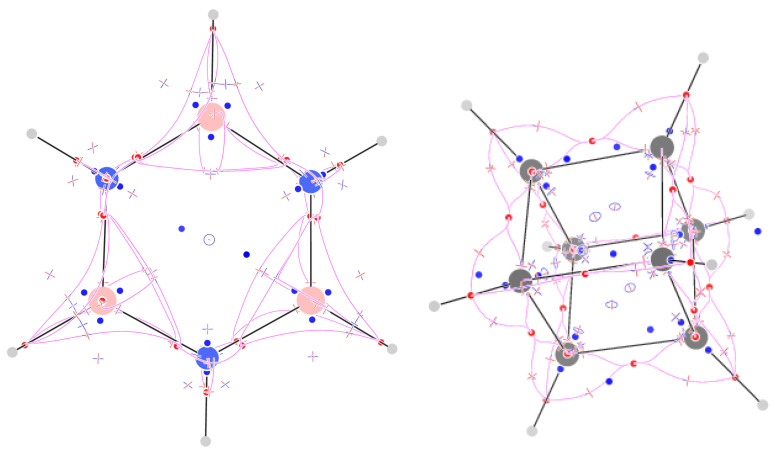
Critical points of $\langle F\rangle$ and trajectories defining the $\langle F\rangle$-graph for borazine (**left**) and cubane (**right**).

The $\langle F\rangle$ field of borazine has 129 isolated CPs (Figure 8). The 24 attractors are located on N and B atoms, on BH and NH bonds (closer to the more electronegative atom), and in pairs, above and below the BN bonds, shifted towards the more electronegative N atom. Around B and N atoms, there are two and three (3,+3) repellers, respectively, and the other two repellers occur above and below the ring, as in benzene and cyclooctatetraene. The 48 (3,−1) CPs are arranged as follows: 1 for each of the six close pairs of π CN bond attractors, 8 around each N atom, and 6 around each B atom. Both atoms have two (3,−1) CPs above and below them, shifted towards the inside of the ring in the case of B atoms. Through any of these CPs, a trajectory passes linking two up or down pairs of π attractors. The pair of π-linking trajectories are slightly on the outside of the ring for N atoms and the inside for B atoms. The inside location of the B trajectories is reminiscent of those of benzene. A second difference between B and N atoms is that, for the B atoms, four CPs occur on the sides of the BH bond and hold four trajectories linking the BH attractor to the π attractors, while for the N atoms, two CPs occur in the vertical plane containing the NH bond. From these CPs, trajectories are also found towards the π maxima, but they do so after being scattered away by the (3,−1) CP located above the N atom. The latter (3,−1) CP also works as a scatterer towards the π attractors for the trajectories starting from the C and N nuclear attractors. The 40 (3,+1) CPs occur 1 at the ring center, 2 and 3 alternating with repellers close to N and B atoms, respectively, and then 3 and 5 around the trajectories of the $\langle F\rangle$ field clustering around B and N atoms and forming topologically square pyramids, one squeezed (for N atoms). The sum of indices is +1. This can be understood as in benzene, in terms of a surface enclosing all but two repellers, which generate two island-like regions of outward flux of $\langle F\rangle$ (see the Supporting Information, Figure S2).

### 2.11. Cubane

The $\langle F\rangle$ field of cubane has 137 CPs (Figure 8). The 28 (3,−3) attractors correspond to the eight C nuclei and the 20 σ bonds. Notably, the (3,−3) CPs of C–C bonds are shifted towards the outer part of the cube, much as happens for the bond paths obtained from ∇ρ [29]. Twenty-three (3,+3) CPs are found: six above the faces, one in the middle of the cube, and two for each C atom, along the diagonal of the cube, one towards the exterior and one towards the interior. The 48 (3,−1) CPs are eight symmetry-equivalent sextets located around C atoms. The distortion of the valence angles from the tetrahedral values is associated with a reduction in the number of CPs, if one compares them with the 10 (3,−1) CPs of methane. The five attractors close to a C atom, if considered as graph vertices, do not form any more a complete graph using (3,−1) CPs as edges: the central attractor on the C atom is connected via (3,−1) CPs to only the three attractors on the C-C bonds, but it is connected to the (3,−1) CH attractor via a (3,+3) CP. The CH attractor is connected to the CC attractors, but there are no (3,+1) CPs on the topological triangle formed by two CC attractors and the CH attractor. Each of the 4=43 topological triangles formed by the C atom and the three CC attractors is associated with a (3,+1) CP. Six more (3,+1) CPs occur on the faces of the cube; they can be thought of as the result of the disappearance of the (3,+1) CPs on the topological triangles, i.e., CH-CC-CC, upon joining the eight CH units forming the molecule. The sum of indices is +5, which can be understood, like in benzene, in terms of a surface enclosing all but six repellers, which generate six island-like regions of outward flux of $\langle F\rangle$ (see the Supporting Information, Figure S2). Inside the surface, the sum of topological indices is, thus, +1−6(+1)=−1, as expected from the topological theory.

## 3. Discussion

The molecules studied in this paper give some insight into the capability of the isotropically averaged Lorentz force density $\langle F\rangle$ contributing to the study of the molecular electronic structure.

As a first point, we note that the attractors of $\langle F\rangle$ tend to be greater in number than the attractors of ∇ρ, where their number corresponds to the number of nuclei (apart from the seldom-observed non-nuclear maxima). In the case of $\langle F\rangle$, the attractors correspond to both nuclei and bonds. In effect, attractors are found very close to H nuclei, only for the ionic Li–H bond, while for B–H, C–H, and N–H bonds the attractors are somewhere along the bonds, closer to the more electronegative atom. In all cases where the electronic structure can be described by core 1s electrons pairs and σ bonds, the total number of attractors $n_{3}$ equals the number of electron pairs (see Table 1). This also holds true for ethylene and cyclooctatetraene, but not for benzene and borazine, showing less (18 vs. 21) and more (24 vs. 21) CPs than the stoichiometric electron pairs, respectively. The case of the triple bond also deserves attention. In acetylene, the number of attractors $n_{3}$ is three less than the number of stoichiometric electron pairs, which hints at the missing triple bond. Then, in propyne, $n_{3}$ matches the number of stoichiometric electron pairs, but in 3-borapropyne $n_{3}$, it is one less than the number of stoichiometric electron pairs. To summarize, although a one-to-one correspondence between electron pairs and attractors holds for saturated molecules, it fails in most cases where bonding can be expected to develop far off of the lines joining the nuclei, or when more than a resonance structure should be considered to describe the electronic structure.

We find it most interesting that C–C bonds of different orders are not characterized by the same (3,−3) CP as for ∇ρ: only for σ bonds do we find a single (3,−3) attractor, while for the double bond of ethylene, we find a (3,−1) saddle flanked by two close attractors, and for the triple bond of acetylene, we find a (3,+1) saddle surrounded by a circle of (2,−2) CPs. Notably, in propyne, the circle of (2,−2) CPs is split into three attractors and three saddles, while the (3,+1) CP is preserved. The appearance of three (3,−3) CPs is a feature of the symmetry of the propyne molecule, as exemplified by the ad hoc studied $C_{2v}$ BH_2_−CCH molecule, where only two (3,−3) CPs are found. However, the (3,+1) CP in the middle of the bond remains, indicating that it is a characteristic feature of the triple bond. The correspondence between bond orders and the number and kind of attractors seems preserved to some extent. In cyclooctatetraene, long and short bonds are associated with one and two attractors, respectively. For the partial bond orders of benzene, we found a single attractor. We note that the possibility to reveal different orders of chemical bonds was noted very early in QCT, in studies of the electron localization function [30] and the Laplacian of the density [23]. To our knowledge, this is the first time that this possibility is highlighted in terms of the magnetic response.

As a second point, when one considers the critical points close to an atom, a certain degree of transferability can be observed, as previously observed for the Laplacian [23]. Indeed, the location and kinds of CPs in ethane and the methyl group of propyne are almost coincident with those of methane. Some transferability is also observed among the critical point found close to the CH_2_ group of ethylene and the CH groups of benzene and cyclooctatetraene. Differences are, instead, observed in the trajectories connecting the attractors on the ring bonds: in benzene these trajectories are shifted towards the inside of the ring, while in cyclooctatetraene they are shifted towards the outside. Notably, in borazine, these trajectories are shifted alternatively inside and outside. This hybrid behavior is well compatible with the main characterization of borazine as a non-aromatic molecule [31], although the different dimensions of the domains of N-centered and B-centered trajectories could comply with some weak aromaticity, which, using the ring current strength was proposed several years ago [32] and recently rediscovered and enriched with considerations based on excited state currents [33].

As a last point, we notice that, for the four molecules studied, which are endowed with a ring structure, the $\langle F\rangle$ field is not always inward-oriented in all places like ∇ρ, but lines of positive (outward) flux of the force were found above and below all rings. In this case, the straightforward expectation of a sum of topological indices equal to -1 is not valid. An adaptation of the algorithm proposed by Leboeuf [28] for counting islands of positive and negative flux of the vector over a sphere surrounding the molecule allows one, in these four cases, to justify the sum of indices. However, in contrast to ref. [28], we find the introduction of asymptotic CPs unnecessary. We find it noteworthy that closed-shell diamagnetic molecules can have narrow spherical angles at large distances where the local magnetic response is paramagnetic.

## 4. Theoretical and Computational Methods

### 4.1. The Magnetically Induced Isotropically Averaged Lorentz Force Density

In the second order in the external magnetic field B, the energy acquired by a freely tumbling isolated closed shell molecule is [34,35]

(1) $$W=-\frac{1}{2}\xi_{iso}B^{2}$$

where the isotropically averaged magnetizability is obtained as one third of the trace of the magnetizability tensor, $\xi_{iso}=\frac{1}{3}\left(\xi_{xx}+\xi_{yy}+\xi_{zz}\right)$, or more briefly, here and in the following, using the Einstein convention of summing over repeated indices, $\xi_{iso}=\frac{1}{3}\xi_{\alpha \alpha }$.

Magnetizability can be computed from the magnetizability density [36,37]

(2) $$\xi_{\mathrm{iso}}=\int \Xi_{\mathrm{iso}}d^{3}r,$$

where [22]

$$\Xi_{\mathrm{iso}}=\frac{\epsilon_{\alpha \beta \gamma }}{6}\left(r_{\beta }-r_{0\beta }\right)J_{\gamma \alpha },$$

with $\epsilon_{\alpha \beta \gamma }$ denoting the Levi-Civita symbol, $r_{\beta }$ denoting a Cartesian displacement from an origin with Cartesian coordinates $r_{0\beta }$, and J denoting the 3×3 current density tensor, which allows the computation of the magnetically induced first-order current density via $J^{B}=JB$. The magnetizability density depends on the arbitrary origin of the coordinates $r_{0}$, so it is not considered a useful tool for QCT. However, by manipulation of the magnetically induced energy [22]:

(3) $$\begin{array}{ccc} W & = & -\frac{B^{2}}{2}\int \Xi_{iso}d^{3}r=-\frac{B^{2}}{12}\int \epsilon_{\alpha \beta \gamma }\left(r_{\beta }-r_{0\beta }\right)J_{\gamma \alpha }d^{3}r \end{array}$$

(4) $$\begin{array}{ccc} & = & -\int {\langle F\rangle }_{\beta }\left(r_{\beta }-r_{0\beta }\right)d^{3}r, \end{array}$$

it can be shown that the isotropically averaged Lorentz force density:

(5) $$\begin{array}{ccc} {\langle F\rangle }_{\beta } & = & \frac{B^{2}}{12}\epsilon_{\beta \gamma \alpha }J_{\gamma \alpha }, \end{array}$$

is an origin-independent vector (and not tensor) field amenable to QCT studies, like ∇ρ. A positive or negative value of the divergence of the isotropically averaged Lorentz force density (DIAL) indicates whether the local contribution to the magnetizability is positive or negative. The usefulness for the study of magnetic aromaticity of π-DIAL (the contributions of π electrons to DIAL) has been recently discussed [38].

Apart from a factor, i.e., DIAL, the divergence of (5) was independently introduced in quantum chemistry by Barquera-Lozada, who called it tpJ(r) [39].

### 4.2. Topological Analysis of the Critical Points

The critical points of a three-dimensional vector field *v* can be classified according to their rank *r* and signature *s* as (r,s) [4], where *r* is the number of non-null eigenvalues of the Jacobian matrix at the critical point, and s=p−n is the difference between the number *p* of positive and the number *n* of negative real components of the eigenvalues of the Jacobian. The sign of the determinant of the Jacobian matrix at the critical point $x^{*}$ is known as its index: ${\mathrm{ind}}_{x^{*}}\left(v\right)=\mathrm{sign}\;\det\;J[v\left(x^{*}\right]={\left(-1\right)}^{n}$ [40]. In appropriate cases, the sum of the indices of the critical points of a vector field must follow the Poincaré–Hopf theorem, which is considered a check of consistency of the topological analysis [28].

**Figure 9:**
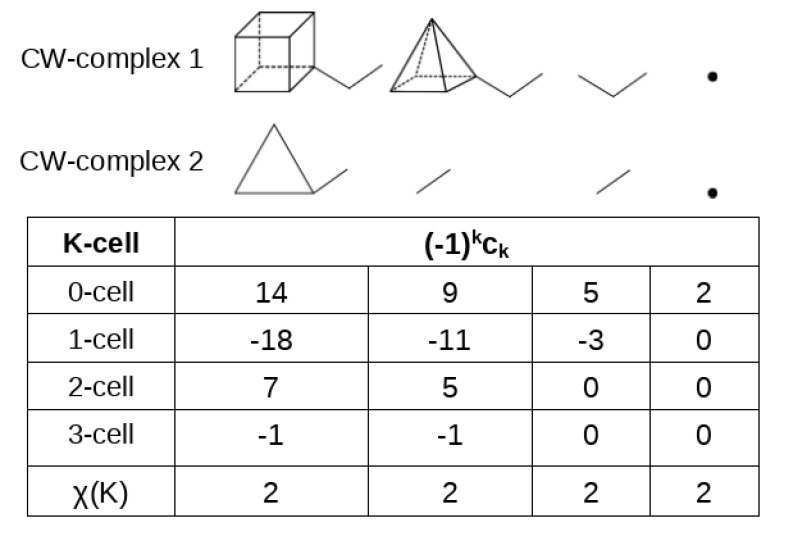
The Euler characteristic of two disconnected CW-complexes is invariant upon contraction of the CW-complexes.

Before discussing this constraint for our field $\langle F\rangle$, we find it useful to introduce the Euler characteristic χ(K) of a CW-complex *K*, which is a collection of *cells* [41]. In our case, it will be sufficient to consider four kinds of cells: 0-cells (isolated points in space), 1-cells (deformable lines connecting two 0-cells), 2-cells (the deformable two-dimensional space contained within a closed loop of 1-cells), and 3-cells (the deformable three-dimensional space contained between two or more 2-cells). The Euler characteristic of the CW-complex χ(K) can be obtained as the difference between even-dimensional cells and odd-dimensional cells:

(6) $$\chi \left(K\right)=\sum_{k}{\left(-1\right)}^{k}c_{k}=c_{0}-c_{1}+c_{2}-c_{3},$$

where $c_{k}$ is the number of *k*-cells. If the CW-complex is compact, it can be contracted to a single point and χ(K)=1. Contraction can be equally performed on a subset of cells of the CW-complex, while leaving the Euler characteristic unchanged. A sketch of the contraction of two CW-complexes, each of them compact, is given in Figure 9.

Equation (6), which, for a single compact CW-complex, can be read as V−E+F−C=1, where *V*, *E*, *F*, and *C* denote the number of vertices, edges, faces, and cages, is consistent with the classical Euler equation V−E+F=2, proposed long ago for a convex polyhedron. Coming back to the critical points of a three-dimensional vector field, we are only interested in full-rank CPs (those with r=3, and, thus, no null eigenvalue of the Jacobian) and Table 2 summarizes their indices.

The four possible full-rank CPs, when ordered for a decreasing number of negative eigenvalues, are (3,−3), (3,−1), (3,1), and (3,3), and will occur in the number $n_{3}$, $n_{2}$, $n_{1}$, $n_{0}$, respectively. In that order, the indices have an alternating sign, so that if the one-to-one correspondence given in column 5 of Table 2 occurs between the critical points of a given kind and the cells of a compact CW-complex ($n_{k}=c_{k}$ for each *k*), Equation (6) can be rewritten as

(7) $$\sum_{k=0}^{3}{\mathrm{ind}}_{k}\left(v\right)n_{k}=1,$$

which matches the three-dimensional Poincaré–Hopf theorem:

(8) $$\sum_{k=0}^{3}{\mathrm{ind}}_{k}\left(v\right)n_{k}=\chi \left(M\right),$$

where χ(M)=1 is the Euler characteristic of the compact three-dimensional manifold, where the vector field is computed. The Euler characteristic χ(M) is an invariant in differential topology, so that it only depends on the manifold *M* and not on the vector field. It is important to notice that the correspondence of Equation (7) with the Poincaré–Hopf theorem only holds because 0-cells correspond to repellers and the vector field turns out to be outward-oriented, which is a hypothesis of the theorem [42]. For a vector field $v^{\prime }$ that is inward-oriented in all places on the boundary of the manifold, the outward orientation can be recovered simply by changing the sign to the field, $v=-v^{\prime }$, which implies a change of the sign of the signature, of all indices, and therefore, for the one-to-one correspondence given in the last column of Table 2 ($n_{3-k}=c_{k}$ for each *k*):

(9) $$\sum_{j=0}^{3}{\mathrm{ind}}_{j}\left(v^{\prime }\right)n_{j}=\sum_{j=0}^{3}{\mathrm{ind}}_{3-j}\left(v^{\prime }\right)n_{3-j}=-\sum_{k=0}^{3}{\mathrm{ind}}_{k}\left(v\right)c_{k}=-1,$$

which, considering that the Euler characteristic of a spherical surface is χ(∂M)=2, can be recognized as a special case of the equation for an inward-oriented field $v^{\prime }$ [43]:

(10) $$\sum_{k=0}^{3}{\mathrm{ind}}_{k}\left(v^{\prime }\right)n_{k}=\chi \left(M\right)-\chi \left(\partial M\right).$$

In our case, the vector field generally has (3,−3) CPs on the outer part of the molecule (an inward-directed field) and, thus, will have $\Sigma {\mathrm{ind}}_{k}n_{k}=-1$. This does not hold when (3,+3) CPs occur on the outer part of the molecule. In that case, the field is partly inward- and partly outward-oriented on a surface enclosing all CPs, and the sum of indices of the isolated CPs, $\Sigma \;{\mathrm{ind}}_{k}n_{k}$ can differ from ±χ(M). In a study of the molecular electrostatic potential (MEP), it was proposed that this difference could be leveled off accounting for asymptotic CPs, which would occur at infinite distance. The presence of these asymptotic CPs was deduced by plotting the MEP on a spherical surface large enough to contain all isolated critical points, and counting the numbers of closed island-like regions of negative and positive values [28]. The adaptation of this procedure also works for the $\langle F\rangle$ field (considering positive and negative values of the flux of $\langle F\rangle$), although our interpretation differs (see Section 2.8).

Equation (8), the celebrated Poincaré–Hopf theorem, assumes that CPs are isolated, although generalizations are possible [43]. In acetylene, we find loops of non-isolated (2,s) CPs. It is proposed that similar cases should be dealt with as symmetry-breaking and then the resulting isolated CPs can be considered [28]. Indeed, one feature of the indices is their stability, which means that small perturbations do not change the sum of the indices of close critical points. A loop of (2,s) CPs can always be thought of as a loop of alternating (2,s−1) and (2,s+1) CPs with a null sum of indices.

### 4.3. Computational Details

Optimized geometries and magnetically perturbed wave functions were obtained using Gaussian 16 [44] and the BHandHLYP functional, which were recently assessed to perform well in the calculation of magnetic properties [45], combined with the pcSseg-4 basis set [46]. Essential parameters of the optimized geometries are given in Table S1.

Critical points were determined by the Newton–Raphson algorithm as implemented in SYSMOIC [26], starting from nuclei and then from points sampled out from a parallelepiped containing the molecule, with a step of 0.1 au, reduced to 0.05 au for molecules with triple bonds, and checking that further reduction did not increase the number of CPs.

The CPs found were sometimes in disagreement with a previous calculation carried out with the smaller 6-31G(d) basis set [22]. We checked that number and kind of critical points were stable upon basis set enlargement by performing a calculation at the aug-pcSseg4 level for benzene, and adding additional basis functions on CPs that were missing in lower level calculations (Table S2). The calculation with additional basis set functions on the (3,+3) CPs above faces in benzene, borazine, cyclooctatetraene, and cubane gave the same unaltered set of CPs. Considering the small size of the molecule studied, all calculations (both geometry optimization and magnetic perturbation) were carried out at the BHandHLYP/pcSseg-4 level.

### 4.4. Graphical Conventions

The critical points are displayed according to the following convention: (3,−3) CPs are small red spheres; (3,+3) CPs are small blue spheres; (3,±1) CPs (with distinguished eigenvalues) are displayed as three crossing segments going along the eigenvectors of the Jacobian, blue for positive eigenvalues, red for negative eigenvalues. In the case of degenerate eigenvalues, a circle is shown in the plane of the eigenvectors corresponding to the degenerate eigenvalues, with the same color code convention: a blue circle for positive degenerate eigenvalues, a red circle for negative degenerate eigenvalues. Once the CPs were found, we computed the trajectories moving out of the (3,−1) CPs along the single positive eigenvalue, to define what we call the $\langle F\rangle$-graph. $\langle F\rangle$-graphs are fairly rich three-dimensional objects; they can be best appreciated when moving interactively, which is possible with the 3D files generated for all of them, using the v3d code, freely distributed at http://sysmoic.chem.unisa.it/MANUAL/ accessed on 8 September 2024. In some instances, the small red spheres corresponding to attractors are covered by the spheres corresponding to atoms in the figures above. This inconvenience can be circumvented by operating on the 3D files attached to the submission with the v3d code. At any rate, the exact number and location of CPs can be always checked with the lists given in the Supporting Information.

## 5. Conclusions

Bader’s concept of defining the molecular structure by a topological characterization of the electron density has generated an enormous amount of activity in experimental and theoretical communities [47,48]. Its extension, known as QCT, addresses different vector fields beyond the gradient of the electron density [1,2,3]. In this framework, the magnetically induced current density, despite the many papers devoted to its topological analysis [49,50,51], remains a kind of outsider, because of its tensorial nature, i.e., it depends on the direction of the inducing field, a piece of information that does not appear in the molecular Hamiltonian. The isotropically averaged Lorentz force density $\langle F\rangle$, which is defined in terms of the current density tensor, offers a new avenue as it is no more a tensor field, it is the average force experienced by a tiny volume within the molecule while the molecule is freely tumbling in three dimensions, and therefore, it does not depend on the orientation of the external field. On continuation of its introduction in quantum chemistry [22], we show here that the topology of $\langle F\rangle$ indeed carries information on the molecular structure: its attractors occur in correspondence with core electrons and chemical bonds. Chemical groups tend to have conservative topological features. CC bonds of a well-defined order are characterized by different CPs: (3,−3) for single bonds, (3,−1) for double bonds, and (3,+1) for triple bonds. The findings of this work highlight the deep connection between QCT and the long-established practice of retrieving information on the molecular structure from the magnetic response. Indeed, both isotropically averaged nuclear shielding and magnetizability can be obtained by the suitable integration of $\langle F\rangle$ [22]. We are confident that further studies on the $\langle F\rangle$ field will prove useful in the study of the molecular electronic structure.

## Figures and Tables

**Table 1:** Numbers $n_{k}$ of isolated critical points with *k* negative eigenvalues and sum of their topological indices ${\mathrm{ind}}_{k}={\left(-1\right)}^{k}$.

Molecule	Formula	-	$n_{3}$	+	$n_{2}$	-	$n_{1}$	+	$n_{0}$	=	$\Sigma_{k}{\mathrm{ind}}_{k}n_{k}$
Lithium hydride	LiH	-	2	+	1	-	0	+	0	=	−1
Methane	CH_4_	-	5	+	10	-	10	+	4	=	−1
Ethane	C_2_H_6_	-	9	+	20	-	20	+	8	=	−1
Ethene	C_2_H_4_	-	8	+	11	-	8	+	4	=	−1
Acetylene	C_2_H_2_	-	4	+	2	-	1	+	2	=	−1
Propyne	C_3_H_4_	-	11	+	21	-	17	+	6	=	−1
3-Borapropyne	BC_2_H_3_	-	9	+	15	-	13	+	6	=	−1
Benzene	C_6_H_6_	-	18	+	36	-	31	+	14	=	+1
Cyclooctateraene	C_8_H_8_	-	28	+	44	-	33	+	18	=	+1
Borazine	B_3_N_3_H_6_	-	24	+	48	-	40	+	17	=	+1
Cubane	C_8_H_8_	-	28	+	48	-	38	+	23	=	+5

**Table 2:** Critical points considered in this paper, and two possible one-to-one correspondences with cells of a CW-complex. *r*, *s*, *n*, and ind are the rank, signature, number of negative eigenvalues, and the topological index.

*r*	*s*	*n*	ind	*k*-Cell	*k*-Cell
3	−3	3	−1	3-cell	0-cell
3	−1	2	1	2-cell	1-cell
3	1	1	−1	1-cell	2-cell
3	3	0	1	0-cell	3-cell
$\Sigma_{j}{\mathrm{ind}}_{j}n_{j}$				1	−1

## Data Availability

The original contributions presented in the study are included in the article/Supplementary Materials. Further inquiries can be directed to the corresponding author.

## Supplementary Materials

The following Supporting Information can be downloaded at: https://www.mdpi.com/article/10.3390/molecules29184502/s1, Table S1. Geometric parameters of the studied molecules. Parameters referred to by 2 letters are bond lengths (bohr). Parameters referred to by 3 letters are bond angles (degrees). Table S2. Isolated critical points of ⟨***F***⟩ of benzene computed at different level of theory. Table S3. Cartesian coordinates, rank, signature and eigenvalues of the Jacobian for the isolated CPs of the ⟨***F***⟩ field computed at the BHandHLYP/pcSseg4//BHandHLYP/pcSseg4 level. For each molecule studied, the number of isolated CPs, and the sum of topological indices is also reported. Figure S1. Critical points of ⟨***F***⟩ and trajectories defining the ⟨***F***⟩—graph for eclipsed ethane. Figure S2. Spheres of 5 au radius containing all isolated CPs of 10 molecules studied in the article. Points on the surface have been coloured in red/blue according to whether the flux of the force, ⟨***F***⟩ · *dS* is outward/inward oriented. From left to right, top to bottom: LiH, CH_4_, C_2_H_6_, C_2_H_4_, C_2_H_2_, C_3_H_5_, BC_2_H_4_, C_8_H_8_ (cubane), C_8_H_8_ (planarized cyclooctatetraene), B_3_N_3_H_6_.

## Abbreviations

The following abbreviations are used in this manuscript:

BCP	Bond Critical Point
CP	Critical Point
DIAL	Divergence of the Isotropically Averaged Lorentz force density
QCT	Quantum Chemical Topology
