# Supplementary material for: A Study of Differential Topology on the Magnetically Induced Isotropically Averaged Lorentz Force Density of a Few Simple Molecules
Source: Molecules. 2024 Sep 23;29(18):4502. doi: 10.3390/molecules29184502 (PMC11435034; doi:10.3390/molecules29184502)
Supplement: Supplementary file 1 [file molecules-29-04502-s001.zip › molecules-3132958-supplementary.pdf]

Article

A Study of Differential Topology on the Magnetically Induced Isotropically Averaged Lorentz Force Density of Few Simple Molecules
Supporting Information

Michele Orza †, **Francesco F. Summa**, **Riccardo Zanasi**, and **Guglielmo Monaco** *

Dipartimento di Chimica e Biologia "Adolfo Zambelli", University of Salerno, Via G. Paolo II, 123, 84184 Fisciano, SA, Italy; michele.orza@unibo.it (M.O.); fsumma@unisa.it (F.F.S.); rzanasi@unisa.it (R.Z.)
* Correspondence: gmonaco@unisa.it
† Current address: Dipartimento di Chimica "Giacomo Ciamician", University of Bologna, Via F. Selmi, 2, 40126 Bologna, BO, Italy.

Table S1. Geometric parameters of the studied molecules. Parameters referred to by 2 letters are bond lengths (bohr). Parameters referred to by 3 letters are bond angles (degrees).

Molecule		Geometric Parameters		Molecule		Geometric Parameters	
LiH	$C_{\infty h}$	LiH	2.99	BH_2CCH	C_2v	CH	1.99
						C≡C	2.26
						BC	2.84
						BH	2.23
CH_4	T_d	CH	2.04	C_6H_6	D_{6h}	CH	2.05
						CC	2.64
C_2H_6	D_{3h}	CH	2.05	C_8H_8	D_{4h}	CH	
		CC	2.89			CH	2.06
		HCC	111.7			CC	2.79
		HCH	107.1			C=C	2.54
C_2H_4	D_{2h}	=CH	2.03	$B_3N_3H_6$	D_{3h}	NH	1.91
		C=C	2.48			BH	2.26
		HC=C	121.7			BN	2.70
		HCH	116.7				
C_2H_2	$D_{\infty h}$	≡CH	1.99	C_8H_8	O_h	CH	2.06
		C≡C	2.24			HCC	2.97
						HCC 125.3	
C_3H_4	C_{3v}	CH	2.04				
		≡CH	1.99				
		HCC≡	110.8				
		HCH	108.1				

Table S2. Isolated critical points of $\langle \boldsymbol{F} \rangle$ of benzene computed at different level of theory.

Level	-	n_3	+	n_2	-	n_1	+	n_0	=	$\Sigma_k \text{ind}_k n_k$
B3LYP/6-31G**//B3LYP/6-31G**	-	18	+	36	-	37	+	12	=	-5
B3LYP/6-311+G(2d,p)//B3LYP/6-31G**	-	18	+	40	-	37	+	14	=	-1
B3LYP/pcSseg-4//B3LYP/6-31G**	-	18	+	36	-	31	+	14	=	1
B3LYP/aug-pcSseg-4//B3LYP/6-31G**	-	18	+	36	-	31	+	14	=	1
B3LYP/aug-pcSseg-4//B3LYP/6-31G**	-	18	+	36	-	31	+	14	=	1
B3LYP/6-31G**// BHandHLYP/pcSseg-2	-	18	+	36	-	31	+	14	=	1
B3LYP/6-31G**// BHandHLYP/pcSseg-4	-	18	+	36	-	31	+	14	=	1
BHandHLYP/aug-pcSseg-4// BHandHLYP/aug-pcSseg-4	-	18	+	36	-	31	+	14	=	1

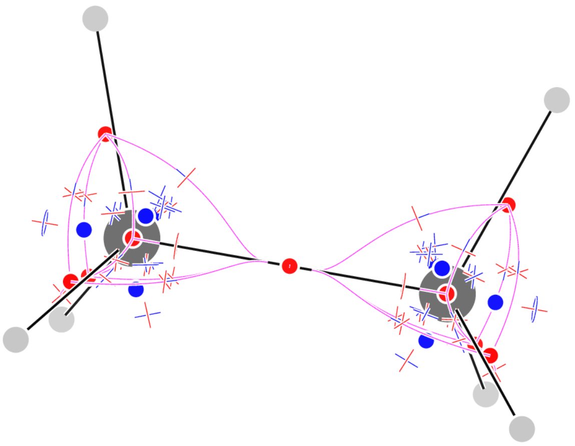

Figure S1. Critical points of $\langle \boldsymbol{F} \rangle$ and trajectories defining the $\langle \boldsymbol{F} \rangle$−graph for eclipsed ethane.

Table S3. Cartesian coordinates, rank, signature and eigenvalues of the Jacobian for the isolated CPs of the $\langle \boldsymbol{F} \rangle$ field computed at the BHandHLYP/pcSseg4//BHandHLYP/pcSseg4 level. For each molecule studied, the number of isolated CPs, and the sum of topological indices is also reported.

LiH							
X	Y	Z	r	s	λ_1	λ_2	λ_3
0.0000	0.0000	0.8078	3	-3	-0.7283	-0.8338	-0.8338
0.0000	0.0000	-2.1753	3	-3	-0.0237	-0.0312	-0.0312
0.0000	0.0000	-0.2030	3	-1	0.0298	-0.0003	-0.0003
$\sum_j n_j = 3$						$\sum_j \mathrm{ind}_j n_j = -1$	

CH_4							
X	Y	Z	r	s	λ_1	λ_2	λ_3
-0.2782	-0.2782	-0.2782	3	3	0.0828	0.0211	0.0211
-0.2782	0.2782	0.2782	3	3	0.0828	0.0211	0.0211
0.2782	-0.2782	0.2782	3	3	0.0828	0.0211	0.0211
0.2782	0.2782	-0.2782	3	3	0.0828	0.0211	0.0211
0.0000	0.4577	0.0000	3	1	0.1222	0.0247	-0.0170
0.4073	-0.4073	0.4073	3	1	0.0166	0.0166	-0.0353
0.0000	0.0000	-0.4577	3	1	0.1222	0.0247	-0.0170
0.4577	0.0000	0.0000	3	1	0.1222	0.0247	-0.0170
0.0000	0.0000	0.4577	3	1	0.1222	0.0247	-0.0170
0.4073	0.4073	-0.4073	3	1	0.0166	0.0166	-0.0353
0.0000	-0.4577	0.0000	3	1	0.1222	0.0247	-0.0170
-0.4577	0.0000	0.0000	3	1	0.1222	0.0247	-0.0170
-0.4073	0.4073	0.4073	3	1	0.0166	0.0166	-0.0353
-0.4073	-0.4073	-0.4073	3	1	0.0166	0.0166	-0.0353
0.0000	0.0000	0.7479	3	-1	0.0244	-0.0115	-0.0425
0.0000	-0.7479	0.0000	3	-1	0.0244	-0.0115	-0.0425
0.7479	0.0000	0.0000	3	-1	0.0244	-0.0115	-0.0425
0.0000	0.7479	0.0000	3	-1	0.0244	-0.0115	-0.0425
0.0000	0.0000	-0.7479	3	-1	0.0244	-0.0115	-0.0425
-0.7479	0.0000	0.0000	3	-1	0.0244	-0.0115	-0.0425
-0.2484	-0.2484	0.2484	3	-1	0.1897	-0.0267	-0.0267
-0.2484	0.2484	-0.2484	3	-1	0.1897	-0.0267	-0.0267
0.2484	-0.2484	-0.2484	3	-1	0.1897	-0.0267	-0.0267
0.2484	0.2484	0.2484	3	-1	0.1897	-0.0267	-0.0267
-0.5357	-0.5357	0.5357	3	-3	-0.0275	-0.0275	-0.0321
-0.5357	0.5357	-0.5357	3	-3	-0.0275	-0.0275	-0.0321
0.5357	0.5357	0.5357	3	-3	-0.0275	-0.0275	-0.0321
0.5357	-0.5357	-0.5357	3	-3	-0.0275	-0.0275	-0.0321
0.0000	0.0000	0.0000	3	-3	-9.5160	-9.5160	-9.5160
$\sum_j n_j = 29$						$\sum_j \mathrm{ind}_j n_j = -1$	

C_2H_6							
X	Y	Z	r	s	λ_1	λ_2	λ_3
-0.3716	-0.2146	-1.3256	3	3	0.1267	0.0235	0.0213
0.0000	-0.4291	1.3256	3	3	0.1267	0.0235	0.0213
0.0000	0.4291	-1.3256	3	3	0.1267	0.0235	0.0213
-0.3716	0.2146	1.3256	3	3	0.1267	0.0235	0.0213
0.0000	0.0000	1.8909	3	3	0.1646	0.0288	0.0288
0.0000	0.0000	-1.8909	3	3	0.1646	0.0288	0.0288
0.3716	-0.2146	-1.3256	3	3	0.1267	0.0235	0.0213
0.3716	0.2146	1.3256	3	3	0.1267	0.0235	0.0213
0.0000	-0.6898	1.1893	3	1	0.0235	0.0151	-0.0407
0.0000	0.6898	-1.1893	3	1	0.0235	0.0151	-0.0407
0.0000	0.0000	2.2591	3	1	0.0154	0.0154	-0.0404

0.0000	0.0000	-2.2591	3	1	0.0154	0.0154	-0.0404
-0.5974	-0.3449	-1.1893	3	1	0.0235	0.0151	-0.0407
-0.5974	0.3449	1.1893	3	1	0.0235	0.0151	-0.0407
-0.3313	-0.1913	1.2542	3	1	0.1615	0.0264	-0.0183
-0.3313	0.1913	-1.2542	3	1	0.1615	0.0264	-0.0183
0.5974	-0.3449	-1.1893	3	1	0.0235	0.0151	-0.0407
0.5974	0.3449	1.1893	3	1	0.0235	0.0151	-0.0407
-0.3305	-0.1908	-1.6385	3	1	0.1904	0.0265	-0.0194
0.0000	-0.3816	1.6385	3	1	0.1904	0.0265	-0.0194
0.3313	0.1913	-1.2542	3	1	0.1615	0.0264	-0.0183
0.3313	-0.1913	1.2542	3	1	0.1615	0.0264	-0.0183
0.0000	0.3816	-1.6385	3	1	0.1904	0.0265	-0.0194
-0.3305	0.1908	1.6385	3	1	0.1904	0.0265	-0.0194
0.0000	-0.3826	-1.2542	3	1	0.1615	0.0264	-0.0183
0.0000	0.3826	1.2542	3	1	0.1615	0.0264	-0.0183
0.3305	-0.1908	-1.6385	3	1	0.1904	0.0265	-0.0194
0.3305	0.1908	1.6385	3	1	0.1904	0.0265	-0.0194
-0.5664	0.3270	-1.0409	3	-1	0.0318	-0.0115	-0.0455
-0.5664	-0.3270	1.0409	3	-1	0.0318	-0.0115	-0.0455
-0.5559	0.3210	1.9760	3	-1	0.0232	-0.0127	-0.0451
-0.5559	-0.3210	-1.9760	3	-1	0.0232	-0.0127	-0.0451
0.0000	-0.6540	-1.0409	3	-1	0.0318	-0.0115	-0.0455
0.0000	0.6540	1.0409	3	-1	0.0318	-0.0115	-0.0455
0.5664	0.3270	-1.0409	3	-1	0.0318	-0.0115	-0.0455
0.5559	0.3210	1.9760	3	-1	0.0232	-0.0127	-0.0451
0.5559	-0.3210	-1.9760	3	-1	0.0232	-0.0127	-0.0451
0.5664	-0.3270	1.0409	3	-1	0.0318	-0.0115	-0.0455
0.0000	0.6419	-1.9760	3	-1	0.0232	-0.0127	-0.0451
0.0000	-0.6419	1.9760	3	-1	0.0232	-0.0127	-0.0451
-0.3464	-0.2000	1.5339	3	-1	0.2411	-0.0263	-0.0265
0.0000	0.0000	-1.0532	3	-1	0.2566	-0.0377	-0.0377
0.0000	0.0000	1.0532	3	-1	0.2566	-0.0377	-0.0377
-0.3464	0.2000	-1.5339	3	-1	0.2411	-0.0263	-0.0265
0.0000	-0.3999	-1.5339	3	-1	0.2411	-0.0263	-0.0265
0.0000	0.3999	1.5339	3	-1	0.2411	-0.0263	-0.0265
0.3464	-0.2000	1.5339	3	-1	0.2411	-0.0263	-0.0265
0.3464	0.2000	-1.5339	3	-1	0.2411	-0.0263	-0.0265
0.0000	-0.9234	-1.8396	3	-3	-0.0274	-0.0291	-0.0308
0.0000	0.9234	1.8396	3	-3	-0.0274	-0.0291	-0.0308
-0.7997	-0.4617	1.8396	3	-3	-0.0274	-0.0291	-0.0308
-0.7997	0.4617	-1.8396	3	-3	-0.0274	-0.0291	-0.0308
0.0000	0.0000	0.0000	3	-3	-0.0040	-0.0328	-0.0328
0.7997	-0.4617	1.8396	3	-3	-0.0274	-0.0291	-0.0308
0.7997	0.4617	-1.8396	3	-3	-0.0274	-0.0291	-0.0308
0.0000	0.0000	1.4500	3	-3	-9.0160	-9.2940	-9.2940
0.0000	0.0000	-1.4500	3	-3	-9.0160	-9.2940	-9.2940
$\sum_j n_j = 57$						$\sum_j \text{ind}_j n_j = -1$	

C_2H_6 Eclipsed							
X	Y	Z	r	s	λ_1	λ_2	λ_3
0.0000	-0.4513	1.3384	3	3	0.1005	0.0210	0.0204
0.0000	-0.4513	-1.3384	3	3	0.1005	0.0210	0.0204
0.0000	0.0000	-1.8976	3	3	0.1569	0.0274	0.0274
0.0000	0.0000	1.8976	3	3	0.1569	0.0274	0.0274
-0.3908	0.2256	-1.3384	3	3	0.1005	0.0210	0.0204
-0.3908	0.2256	1.3384	3	3	0.1005	0.0210	0.0204
0.3908	0.2256	1.3384	3	3	0.1005	0.0210	0.0204

0.3908	0.2256	-1.3384	3	3	0.1005	0.0210	0.0204
0.0000	0.0000	-2.2584	3	1	0.0143	0.0143	-0.0399
0.0000	0.0000	2.2584	3	1	0.0143	0.0143	-0.0399
-0.5722	0.3304	1.1937	3	1	0.0241	0.0140	-0.0376
-0.5722	0.3304	-1.1937	3	1	0.0241	0.0140	-0.0376
0.0000	-0.6608	-1.1937	3	1	0.0241	0.0140	-0.0376
0.0000	-0.6608	1.1937	3	1	0.0241	0.0140	-0.0376
0.0000	0.4067	1.2677	3	1	0.1327	0.0250	-0.0176
0.0000	0.4067	-1.2677	3	1	0.1327	0.0250	-0.0176
-0.3522	-0.2033	-1.2677	3	1	0.1327	0.0250	-0.0176
-0.3522	-0.2033	1.2677	3	1	0.1327	0.0250	-0.0176
0.5722	0.3304	-1.1937	3	1	0.0241	0.0140	-0.0376
0.0000	-0.3976	-1.6346	3	1	0.1662	0.0256	-0.0171
0.0000	-0.3976	1.6346	3	1	0.1662	0.0256	-0.0171
0.5722	0.3304	1.1937	3	1	0.0241	0.0140	-0.0376
-0.3443	0.1988	-1.6346	3	1	0.1662	0.0256	-0.0171
-0.3443	0.1988	1.6346	3	1	0.1662	0.0256	-0.0171
0.3522	-0.2033	-1.2677	3	1	0.1327	0.0250	-0.0176
0.3522	-0.2033	1.2677	3	1	0.1327	0.0250	-0.0176
0.3443	0.1988	-1.6346	3	1	0.1662	0.0256	-0.0171
0.3443	0.1988	1.6346	3	1	0.1662	0.0256	-0.0171
-0.5434	-0.3137	1.0627	3	-1	0.0331	-0.0104	-0.0438
-0.5434	-0.3137	-1.0627	3	-1	0.0331	-0.0104	-0.0438
0.0000	0.6275	-1.0627	3	-1	0.0331	-0.0104	-0.0438
0.0000	0.6275	1.0627	3	-1	0.0331	-0.0104	-0.0438
-0.5280	0.3048	-1.9926	3	-1	0.0225	-0.0123	-0.0448
-0.5280	0.3048	1.9926	3	-1	0.0225	-0.0123	-0.0448
-0.3575	-0.2064	1.5305	3	-1	0.2138	-0.0237	-0.0254
-0.3575	-0.2064	-1.5305	3	-1	0.2138	-0.0237	-0.0254
0.0000	-0.6096	1.9926	3	-1	0.0225	-0.0123	-0.0448
0.0000	-0.6096	-1.9926	3	-1	0.0225	-0.0123	-0.0448
0.5434	-0.3137	1.0627	3	-1	0.0331	-0.0104	-0.0438
0.5434	-0.3137	-1.0627	3	-1	0.0331	-0.0104	-0.0438
0.5280	0.3048	-1.9926	3	-1	0.0225	-0.0123	-0.0448
0.5280	0.3048	1.9926	3	-1	0.0225	-0.0123	-0.0448
0.3575	-0.2064	1.5305	3	-1	0.2138	-0.0237	-0.0254
0.3575	-0.2064	-1.5305	3	-1	0.2138	-0.0237	-0.0254
0.0000	0.4128	-1.5305	3	-1	0.2138	-0.0237	-0.0254
0.0000	0.4128	1.5305	3	-1	0.2138	-0.0237	-0.0254
0.0000	0.0000	1.0455	3	-1	0.2338	-0.0411	-0.0411
0.0000	0.0000	-1.0455	3	-1	0.2338	-0.0411	-0.0411
-0.7708	-0.4450	1.8551	3	-3	-0.0271	-0.0286	-0.0315
-0.7708	-0.4450	-1.8551	3	-3	-0.0271	-0.0286	-0.0315
0.0000	0.0000	0.0000	3	-3	-0.0030	-0.0329	-0.0329
0.0000	0.8901	1.8551	3	-3	-0.0271	-0.0286	-0.0315
0.0000	0.8901	-1.8551	3	-3	-0.0271	-0.0286	-0.0315
0.7708	-0.4450	1.8551	3	-3	-0.0271	-0.0286	-0.0315
0.7708	-0.4450	-1.8551	3	-3	-0.0271	-0.0286	-0.0315
0.0000	0.0000	-1.4480	3	-3	-8.7110	-9.2110	-9.2110
0.0000	0.0000	1.4480	3	-3	-8.7110	-9.2110	-9.2110
$\sum_j n_j = 57$						$\sum_j \text{ind}_j n_j = -1$	

C_2H_4							
X	Y	Z	r	s	λ_1	λ_2	λ_3
0.0000	0.3382	-1.1932	3	3	0.4266	0.2122	0.1245
0.0000	0.3382	1.1932	3	3	0.4266	0.2122	0.1245
0.0000	-0.3382	-1.1932	3	3	0.4266	0.2122	0.1245

0.0000	-0.3382	1.1932	3	3	0.4266	0.2122	0.1245
0.0000	0.0000	-2.3128	3	1	0.0223	0.0055	-0.0342
0.0000	0.0000	2.3128	3	1	0.0223	0.0055	-0.0342
0.0000	0.9484	-1.0340	3	1	0.0318	0.0153	-0.0430
0.0000	0.9484	1.0340	3	1	0.0318	0.0153	-0.0430
0.0000	-0.9484	-1.0340	3	1	0.0318	0.0153	-0.0430
0.0000	-0.9484	1.0340	3	1	0.0318	0.0153	-0.0430
0.0000	0.0000	-0.9556	3	1	0.9249	0.3227	-0.1477
0.0000	0.0000	0.9556	3	1	0.9249	0.3227	-0.1477
-0.9575	0.7358	1.5659	3	-1	0.0078	-0.0064	-0.0221
-0.9575	0.7358	-1.5659	3	-1	0.0078	-0.0064	-0.0221
-0.9575	-0.7358	1.5659	3	-1	0.0078	-0.0064	-0.0221
-0.9575	-0.7358	-1.5659	3	-1	0.0078	-0.0064	-0.0221
0.9575	-0.7358	-1.5659	3	-1	0.0078	-0.0064	-0.0221
0.9575	-0.7358	1.5659	3	-1	0.0078	-0.0064	-0.0221
0.9575	0.7358	-1.5659	3	-1	0.0078	-0.0064	-0.0221
0.9575	0.7358	1.5659	3	-1	0.0078	-0.0064	-0.0221
0.0000	0.0000	0.0000	3	-1	0.0060	-0.0427	-0.0767
0.0000	0.0000	-1.4936	3	-1	0.6087	-0.1446	-0.1646
0.0000	0.0000	1.4936	3	-1	0.6087	-0.1446	-0.1646
0.0000	-1.1417	-1.9547	3	-3	-0.0176	-0.0204	-0.0313
0.0000	-1.1417	1.9547	3	-3	-0.0176	-0.0204	-0.0313
0.0000	1.1417	-1.9547	3	-3	-0.0176	-0.0204	-0.0313
0.0000	1.1417	1.9547	3	-3	-0.0176	-0.0204	-0.0313
-0.3670	0.0000	0.0000	3	-3	-0.0095	-0.0386	-0.0586
0.3670	0.0000	0.0000	3	-3	-0.0095	-0.0386	-0.0586
0.0000	0.0000	-1.2823	3	-3	-4.0880	-5.3220	-5.3970
0.0000	0.0000	1.2823	3	-3	-4.0880	-5.3220	-5.3970
$\sum_j n_j = 31$						$\sum_j \mathrm{ind}_j n_j = -1$	

C_2H_2							
X	Y	Z	r	s	λ_1	λ_2	λ_3
0.0000	0.0000	-0.8353	3	3	1.0800	0.0165	0.0165
0.0000	0.0000	0.8353	3	3	1.0800	0.0165	0.0165
0.0000	0.0000	0.0000	3	1	0.0018	0.0018	-0.1278
0.0000	0.0000	-1.3056	3	-1	0.5066	-1.0090	-1.0090
0.0000	0.0000	1.3056	3	-1	0.5066	-1.0090	-1.0090
0.0000	0.0000	-2.4323	3	-3	-0.0261	-0.0286	-0.0286
0.0000	0.0000	2.4323	3	-3	-0.0261	-0.0286	-0.0286
0.0000	0.0000	-1.2090	3	-3	-1.1940	-3.3590	-3.3590
0.0000	0.0000	1.2090	3	-3	-1.1940	-3.3590	-3.3590
$\sum_j n_j = 9$						$\sum_j \mathrm{ind}_j n_j = -1$	

C_3H_4							
X	Y	Z	r	s	λ_1	λ_2	λ_3
0.0000	0.0000	0.7430	3	3	1.1490	0.0054	0.0054
0.0000	-0.4272	-2.2926	3	3	0.1259	0.0271	0.0223
-0.3699	0.2136	-2.2926	3	3	0.1259	0.0271	0.0223
0.0000	0.0000	2.4499	3	3	1.0400	0.0308	0.0308
0.3699	0.2136	-2.2926	3	3	0.1259	0.0271	0.0223
0.0000	0.0000	-2.8629	3	3	0.1821	0.0092	0.0092
0.0000	0.8340	2.9693	3	1	0.0324	0.0001	-0.0423
0.0000	0.0000	1.6262	3	1	0.0025	0.0025	-0.1239
-0.7284	-0.4205	0.2656	3	1	0.0410	0.0007	-0.0432
0.0000	0.8411	0.2656	3	1	0.0410	0.0007	-0.0432
0.0000	0.0000	-3.2529	3	1	0.0112	0.0112	-0.0384
0.0000	-0.7102	-2.2253	3	1	0.0177	0.0171	-0.0391

-0.6150	0.3551	-2.2253	3	1	0.0177	0.0171	-0.0391
0.7284	-0.4205	0.2656	3	1	0.0410	0.0007	-0.0432
-0.3088	-0.1783	-2.2073	3	1	0.1826	0.0308	-0.0183
-0.7223	-0.4170	2.9693	3	1	0.0324	0.0001	-0.0423
0.7223	-0.4170	2.9693	3	1	0.0324	0.0001	-0.0423
0.0000	0.3566	-2.2073	3	1	0.1826	0.0308	-0.0183
0.6150	0.3551	-2.2253	3	1	0.0177	0.0171	-0.0391
0.1730	0.0999	-2.8098	3	1	0.1899	0.0167	-0.0102
0.3088	-0.1783	-2.2073	3	1	0.1826	0.0308	-0.0183
-0.1730	0.0999	-2.8098	3	1	0.1899	0.0167	-0.0102
0.0000	-0.1998	-2.8098	3	1	0.1899	0.0167	-0.0102
-0.7227	0.4172	2.9707	3	-1	0.0323	-0.0001	-0.0423
0.0000	-0.8344	2.9707	3	-1	0.0323	-0.0001	-0.0423
-0.1634	-0.0944	1.6269	3	-1	0.0002	-0.0046	-0.1144
-0.7337	0.4236	0.2624	3	-1	0.0397	-0.0007	-0.0423
0.0000	-0.8472	0.2624	3	-1	0.0397	-0.0007	-0.0423
0.0000	0.6843	-2.0102	3	-1	0.0263	-0.0114	-0.0427
-0.4887	0.2822	-3.0091	3	-1	0.0228	-0.0095	-0.0438
0.4887	0.2822	-3.0091	3	-1	0.0228	-0.0095	-0.0438
0.5927	-0.3422	-2.0102	3	-1	0.0263	-0.0114	-0.0427
0.0000	-0.5643	-3.0091	3	-1	0.0228	-0.0095	-0.0438
-0.5927	-0.3422	-2.0102	3	-1	0.0263	-0.0114	-0.0427
0.0000	0.0000	-2.0477	3	-1	0.2881	-0.0322	-0.0322
0.0000	0.0000	0.2906	3	-1	0.5489	-1.0850	-1.0850
0.7337	0.4236	0.2624	3	-1	0.0397	-0.0007	-0.0423
-0.2819	-0.1628	-2.6817	3	-1	0.2420	-0.0209	-0.0267
0.7227	0.4172	2.9707	3	-1	0.0323	-0.0001	-0.0423
0.0000	0.0000	2.9323	3	-1	0.5174	-0.9026	-0.9026
0.2819	-0.1628	-2.6817	3	-1	0.2420	-0.0209	-0.0267
0.1634	-0.0944	1.6269	3	-1	0.0002	-0.0046	-0.1144
0.0000	0.3255	-2.6817	3	-1	0.2420	-0.0209	-0.0267
0.0000	0.1887	1.6269	3	-1	0.0002	-0.0046	-0.1144
0.0000	0.0000	4.0472	3	-3	-0.0249	-0.0272	-0.0272
-0.7929	-0.4578	-2.8111	3	-3	-0.0260	-0.0286	-0.0295
0.1674	0.0966	1.6269	3	-3	-0.0002	-0.0047	-0.1139
0.0000	0.9156	-2.8111	3	-3	-0.0260	-0.0286	-0.0295
0.7929	-0.4578	-2.8111	3	-3	-0.0260	-0.0286	-0.0295
0.0000	0.0000	-1.2941	3	-3	-0.0215	-0.0262	-0.0262
0.0000	-0.1932	1.6269	3	-3	-0.0002	-0.0047	-0.1139
-0.1674	0.0966	1.6269	3	-3	-0.0002	-0.0047	-0.1139
0.0000	0.0000	0.3829	3	-3	-1.2470	-3.4740	-3.4740
0.0000	0.0000	2.8250	3	-3	-1.3450	-3.4560	-3.4560
0.0000	0.0000	-2.4441	3	-3	-9.2990	-9.5400	-9.5400
$\sum_j n_j = 55$						$\sum_j \text{ind}_j n_j = -1$	

BH_2CCH							
0.0000	0.0000	-3.0413	3	3	0.2435	0.1038	0.0572
0.3594	0.0000	0.2560	3	3	0.4070	0.1667	0.0042
0.0000	-0.2605	-2.3280	3	3	0.2784	0.0806	0.0200
0.0000	0.2605	-2.3280	3	3	0.2784	0.0806	0.0200
-0.3594	0.0000	0.2560	3	3	0.4070	0.1667	0.0042
0.0000	0.0000	2.1942	3	3	1.1350	0.1988	0.1806
0.0000	0.9893	2.8951	3	1	0.0155	0.0013	-0.0326
0.0000	-1.1696	-1.9548	3	1	0.0177	0.0058	-0.0161
0.0000	1.1696	-1.9548	3	1	0.0177	0.0058	-0.0161
0.0000	-0.9893	2.8951	3	1	0.0155	0.0013	-0.0326
0.0000	0.3588	-2.6992	3	1	0.3636	0.0764	-0.0436

0.0000	0.0000	-3.9489	3	1	0.0176	0.0078	-0.0154
0.0000	-0.3588	-2.6992	3	1	0.3636	0.0764	-0.0436
0.0000	0.0000	1.3595	3	1	0.0139	0.0123	-0.1306
0.0000	-0.8423	0.0871	3	1	0.0446	0.0038	-0.0459
0.0000	0.3168	0.3289	3	1	0.5567	0.1128	-0.0172
0.0000	-0.3168	0.3289	3	1	0.5567	0.1128	-0.0172
0.0000	0.8423	0.0871	3	1	0.0446	0.0038	-0.0459
0.0000	0.0000	-2.2390	3	1	0.3213	0.0756	-0.0140
-1.0335	0.0000	3.0097	3	-1	0.0124	-0.0010	-0.0248
-2.1103	-1.1881	-2.3477	3	-1	0.0010	-0.0007±i	0.0001
-2.1103	1.1881	-2.3477	3	-1	0.0010	-0.0007±i	0.0001
2.1103	-1.1881	-2.3477	3	-1	0.0010	-0.0007±i	0.0001
2.1103	1.1881	-2.3477	3	-1	0.0010	-0.0007±i	0.0001
1.0335	0.0000	3.0097	3	-1	0.0124	-0.0010	-0.0248
0.8656	0.0000	0.0471	3	-1	0.0388	-0.0039	-0.0431
-0.8656	0.0000	0.0471	3	-1	0.0388	-0.0039	-0.0431
0.0000	0.0000	0.4645	3	-1	1.1990	-0.1060	-0.1974
0.0000	0.4528	1.3638	3	-1	0.0002	-0.0187	-0.0842
0.0000	-0.4528	1.3638	3	-1	0.0002	-0.0187	-0.0842
0.0000	0.0000	2.6335	3	-1	0.3613	-1.0380	-1.1460
-0.3018	0.0000	-2.5764	3	-1	0.5067	-0.1004	-0.1269
0.0000	0.0000	0.0124	3	-1	0.8530	-0.7217	-0.7842
0.3018	0.0000	-2.5764	3	-1	0.5067	-0.1004	-0.1269
0.0000	0.0000	2.5793	3	-3	-0.5805	-2.2610	-2.4080
0.0000	0.0000	0.1717	3	-3	-3.4930	-5.7550	-5.9390
0.0000	0.0000	-2.6187	3	-3	-4.6050	-4.8790	-4.8930
0.0000	1.7635	-3.6131	3	-3	-0.0232	-0.0273	-0.0370
0.0000	-1.7635	-3.6131	3	-3	-0.0232	-0.0273	-0.0370
0.4697	0.0000	1.3623	3	-3	-0.0003	-0.0164	-0.0833
0.0000	0.0000	-0.9976	3	-3	-0.0084	-0.0233	-0.0466
-0.4697	0.0000	1.3623	3	-3	-0.0003	-0.0164	-0.0833
0.0000	0.0000	3.8271	3	-3	-0.0236	-0.0255	-0.0258
$\sum_j n_j = 43$						$\sum_j \mathrm{ind}_j n_j = -1$	

C_6H_6							
0.0000	2.6241	0.0000	3	-3	-6.9970	-7.0380	-7.1080
0.0000	0.0000	-1.8676	3	3	0.0013	0.0013	0.0006
0.0000	-0.0000	1.8676	3	3	0.0013	0.0013	0.0006
1.9676	1.1360	0.0000	3	3	0.4801	0.2794	0.1180
1.9676	-1.1360	0.0000	3	3	0.4801	0.2794	0.1180
-2.5261	-1.4584	0.0000	3	3	0.5263	0.1067	0.0412
-2.5261	1.4584	0.0000	3	3	0.5263	0.1067	0.0412
-1.9676	-1.1360	0.0000	3	3	0.4801	0.2794	0.1180
-1.9676	1.1360	0.0000	3	3	0.4801	0.2794	0.1180
0.0000	-2.2720	0.0000	3	3	0.4801	0.2794	0.1180
0.0000	2.2720	0.0000	3	3	0.4801	0.2794	0.1180
0.0000	-2.9168	0.0000	3	3	0.5263	0.1067	0.0412
0.0000	2.9168	0.0000	3	3	0.5263	0.1067	0.0412
2.5261	-1.4584	0.0000	3	3	0.5263	0.1067	0.0412
2.5261	1.4584	0.0000	3	3	0.5263	0.1067	0.0412
-2.4609	-2.3147	0.0000	3	1	0.0275	0.0100	-0.0386
-2.4609	2.3147	0.0000	3	1	0.0275	0.0100	-0.0386
-3.2351	-0.9739	0.0000	3	1	0.0275	0.0100	-0.0386
0.2726	-2.5611	0.0000	3	1	0.7760	0.1677	-0.1187
0.2726	2.5611	0.0000	3	1	0.7760	0.1677	-0.1187
-3.2351	0.9739	0.0000	3	1	0.0275	0.0100	-0.0386
0.0000	0.0000	0.0000	3	1	0.0061	0.0061	-0.0017

-2.3542	-1.0445	0.0000	3	1	0.7760	0.1677	-0.1187
-0.7741	-3.2886	0.0000	3	1	0.0275	0.0100	-0.0386
-1.4184	0.8189	0.0000	3	1	0.0402	0.0196	-0.0447
-1.4184	-0.8189	0.0000	3	1	0.0402	0.0196	-0.0447
-0.7741	3.2886	0.0000	3	1	0.0275	0.0100	-0.0386
-2.3542	1.0445	0.0000	3	1	0.7760	0.1677	-0.1187
-2.0817	-1.5166	0.0000	3	1	0.7760	0.1677	-0.1187
-2.0817	1.5166	0.0000	3	1	0.7760	0.1677	-0.1187
0.7741	3.2886	0.0000	3	1	0.0275	0.0100	-0.0386
0.0000	-1.6378	0.0000	3	1	0.0402	0.0196	-0.0447
0.0000	1.6378	-0.0000	3	1	0.0402	0.0196	-0.0447
0.7741	-3.2886	0.0000	3	1	0.0275	0.0100	-0.0386
2.4609	-2.3147	0.0000	3	1	0.0275	0.0100	-0.0386
2.4609	2.3147	0.0000	3	1	0.0275	0.0100	-0.0386
1.4184	-0.8189	0.0000	3	1	0.0402	0.0196	-0.0447
1.4184	0.8189	0.0000	3	1	0.0402	0.0196	-0.0447
-0.2726	-2.5611	0.0000	3	1	0.7760	0.1677	-0.1187
-0.2726	2.5611	0.0000	3	1	0.7760	0.1677	-0.1187
3.2351	-0.9739	0.0000	3	1	0.0275	0.0100	-0.0386
3.2351	0.9739	0.0000	3	1	0.0275	0.0100	-0.0386
2.0817	-1.5166	0.0000	3	1	0.7760	0.1677	-0.1187
2.0817	1.5166	0.0000	3	1	0.7760	0.1677	-0.1187
2.3542	-1.0445	0.0000	3	1	0.7760	0.1677	-0.1187
2.3542	1.0445	0.0000	3	1	0.7760	0.1677	-0.1187
-3.0228	-1.7452	0.8256	3	-1	0.0100	-0.0072	-0.0260
-3.0228	-1.7452	-0.8256	3	-1	0.0100	-0.0072	-0.0260
-3.0228	1.7452	0.8256	3	-1	0.0100	-0.0072	-0.0260
-3.0228	1.7452	-0.8256	3	-1	0.0100	-0.0072	-0.0260
-1.5218	-0.8786	-0.9539	3	-1	0.0094	-0.0121	-0.0177
-1.5218	-0.8786	0.9539	3	-1	0.0094	-0.0121	-0.0177
-1.5218	0.8786	-0.9539	3	-1	0.0094	-0.0121	-0.0177
-1.5218	0.8786	0.9539	3	-1	0.0094	-0.0121	-0.0177
-2.3658	-1.3659	-0.2124	3	-1	0.8851	-0.1209	-0.2214
-2.3658	-1.3659	0.2124	3	-1	0.8851	-0.1209	-0.2214
-2.3658	1.3659	-0.2124	3	-1	0.8851	-0.1209	-0.2214
-2.3658	1.3659	0.2124	3	-1	0.8851	-0.1209	-0.2214
3.0228	1.7452	-0.8256	3	-1	0.0100	-0.0072	-0.0260
3.0228	1.7452	0.8256	3	-1	0.0100	-0.0072	-0.0260
3.0228	-1.7452	-0.8256	3	-1	0.0100	-0.0072	-0.0260
3.0228	-1.7452	0.8256	3	-1	0.0100	-0.0072	-0.0260
0.0000	-3.4904	-0.8256	3	-1	0.0100	-0.0072	-0.0260
0.0000	-1.7573	-0.9539	3	-1	0.0094	-0.0121	-0.0177
0.0000	-1.7573	0.9539	3	-1	0.0094	-0.0121	-0.0177
0.0000	-3.4904	0.8256	3	-1	0.0100	-0.0072	-0.0260
0.0000	3.4904	-0.8256	3	-1	0.0100	-0.0072	-0.0260
0.0000	1.7573	-0.9539	3	-1	0.0094	-0.0121	-0.0177
0.0000	1.7573	0.9539	3	-1	0.0094	-0.0121	-0.0177
0.0000	3.4904	0.8256	3	-1	0.0100	-0.0072	-0.0260
1.5218	0.8786	-0.9539	3	-1	0.0094	-0.0121	-0.0177
1.5218	0.8786	0.9539	3	-1	0.0094	-0.0121	-0.0177
1.5218	-0.8786	-0.9539	3	-1	0.0094	-0.0121	-0.0177
1.5218	-0.8786	0.9539	3	-1	0.0094	-0.0121	-0.0177
0.0000	-2.7318	-0.2124	3	-1	0.8851	-0.1209	-0.2214
0.0000	-2.7318	0.2124	3	-1	0.8851	-0.1209	-0.2214
0.0000	2.7318	-0.2124	3	-1	0.8851	-0.1209	-0.2214
0.0000	2.7318	0.2124	3	-1	0.8851	-0.1209	-0.2214
2.3658	-1.3659	-0.2124	3	-1	0.8851	-0.1209	-0.2214

2.3658	-1.3659	0.2124	3	-1	0.8851	-0.1209	-0.2214
2.3658	1.3659	-0.2124	3	-1	0.8851	-0.1209	-0.2214
2.3658	1.3659	0.2124	3	-1	0.8851	-0.1209	-0.2214
-2.2726	1.3121	0.0000	3	-3	-6.9970	-7.0380	-7.1080
0.0000	-2.6241	0.0000	3	-3	-6.9970	-7.0380	-7.1080
-2.2726	-1.3121	0.0000	3	-3	-6.9970	-7.0380	-7.1080
2.2726	-1.3121	0.0000	3	-3	-6.9970	-7.0380	-7.1080
2.2726	1.3121	0.0000	3	-3	-6.9970	-7.0380	-7.1080
0.0000	3.9290	0.0000	3	-3	-0.0168	-0.0205	-0.0310
-3.4026	1.9645	0.0000	3	-3	-0.0168	-0.0205	-0.0310
0.0000	-3.9290	0.0000	3	-3	-0.0168	-0.0205	-0.0310
-3.4026	-1.9645	0.0000	3	-3	-0.0168	-0.0205	-0.0310
3.4026	-1.9645	0.0000	3	-3	-0.0168	-0.0205	-0.0310
3.4026	1.9645	0.0000	3	-3	-0.0168	-0.0205	-0.0310
-2.2927	0.0000	0.0000	3	-3	-0.0007	-0.0363	-0.0508
2.2927	0.0000	0.0000	3	-3	-0.0007	-0.0363	-0.0508
-1.1464	-1.9855	0.0000	3	-3	-0.0007	-0.0363	-0.0508
-1.1464	1.9855	0.0000	3	-3	-0.0007	-0.0363	-0.0508
1.1464	-1.9855	0.0000	3	-3	-0.0007	-0.0363	-0.0508
1.1464	1.9855	0.0000	3	-3	-0.0007	-0.0363	-0.0508
$\sum_j n_j = 99$						$\sum_j \text{ind}_j n_j = 1$	

$B_3H_6N_3$							
X	Y	Z	r	s	λ_1	λ_2	λ_3
2.3558	1.3601	0.0000	3	-3	-4.9900	-5.0550	-5.1220
-0.0000	2.6485	0.0000	3	-3	-12.8000	-13.5700	-13.9000
-2.3558	1.3601	0.0000	3	-3	-4.9900	-5.0550	-5.1220
-2.2937	-1.3242	0.0000	3	-3	-12.8000	-13.5700	-13.9000
0.0000	-2.7202	0.0000	3	-3	-4.9900	-5.0550	-5.1220
2.2937	-1.3242	0.0000	3	-3	-12.8000	-13.5700	-13.9000
4.0270	2.3250	0.0000	3	-3	-0.0213	-0.0251	-0.0327
0.0000	-1.4566	0.0000	3	1	0.0273	0.0031	-0.0159
-4.0270	2.3250	0.0000	3	-3	-0.0213	-0.0251	-0.0327
1.2614	0.7283	0.0000	3	1	0.0273	0.0031	-0.0159
0.0000	-4.6500	0.0000	3	-3	-0.0213	-0.0251	-0.0327
-1.2614	0.7283	0.0000	3	1	0.0273	0.0031	-0.0159
0.0000	0.0000	0.0000	3	1	0.0049	0.0049	-0.0011
-0.2285	-3.3534	-1.6163	3	-1	0.0029	-0.0001	-0.0045
-0.2285	-3.3534	1.6163	3	-1	0.0029	-0.0001	-0.0045
0.2285	-3.3534	-1.6163	3	-1	0.0029	-0.0001	-0.0045
0.2285	-3.3534	1.6163	3	-1	0.0029	-0.0001	-0.0045
0.0000	3.3513	-0.3170	3	-1	0.0069	-0.0128	-0.0742
0.0000	3.3513	0.3170	3	-1	0.0069	-0.0128	-0.0742
-2.2630	-1.3066	-0.8748	3	-1	0.0007	-0.0144	-0.0751
-2.2630	-1.3066	0.8748	3	-1	0.0007	-0.0144	-0.0751
-3.0255	-1.2591	0.0000	3	1	0.0241	0.0120	-0.0863
-2.5629	-1.4797	0.0000	3	3	0.4992	0.1480	0.1450
-1.3257	0.7654	-0.8679	3	-1	0.0157	-0.0030	-0.0090
-1.3257	0.7654	0.8679	3	-1	0.0157	-0.0030	-0.0090
-3.0184	1.4788	-1.6163	3	-1	0.0029	-0.0001	-0.0045
-2.8916	1.6695	-1.6560	3	1	0.0026	0.0001	-0.0043
-2.7898	1.8746	-1.6163	3	-1	0.0029	-0.0001	-0.0045
-2.7898	1.8746	1.6163	3	-1	0.0029	-0.0001	-0.0045
-2.8916	1.6695	1.6560	3	1	0.0026	0.0001	-0.0043
-3.0184	1.4788	1.6163	3	-1	0.0029	-0.0001	-0.0045
-1.9996	1.5467	0.0000	3	1	0.3133	0.0391	-0.0384
0.0000	2.6131	-0.8748	3	-1	0.0007	-0.0144	-0.0751

0.0000	2.6131	0.8748	3	-1	0.0007	-0.0144	-0.0751
0.0000	0.0000	-4.1618	3	3	0.0000	0.0000	0.0000
0.0000	0.0000	4.1618	3	3	0.0000	0.0000	0.0000
-1.6054	-1.8274	0.0000	3	-1	0.0011	-0.0384	-0.0827
-3.4990	0.7847	0.0000	3	1	0.0200	0.0033	-0.0144
1.3257	0.7654	-0.8679	3	-1	0.0157	-0.0030	-0.0090
1.3257	0.7654	0.8679	3	-1	0.0157	-0.0030	-0.0090
1.7288	-0.9981	0.0000	3	1	0.0605	0.0576	-0.0942
-2.4291	2.6379	0.0000	3	1	0.0200	0.0033	-0.0144
3.4990	0.7847	0.0000	3	1	0.0200	0.0033	-0.0144
-2.3994	1.3853	-0.3639	3	-1	0.3315	-0.0476	-0.0509
-2.3994	1.3853	0.3639	3	-1	0.3315	-0.0476	-0.0509
0.4224	3.2497	0.0000	3	1	0.0241	0.0120	-0.0863
-2.9603	-1.7091	0.0000	3	-3	-0.0051	-0.0204	-0.0655
-2.3393	0.9583	0.0000	3	1	0.3133	0.0391	-0.0384
3.0184	1.4788	1.6163	3	-1	0.0029	-0.0001	-0.0045
3.0184	1.4788	-1.6163	3	-1	0.0029	-0.0001	-0.0045
-0.7312	2.3256	-0.3153	3	-3	-0.0016	-0.0351	-0.0785
-0.7312	2.3256	0.3153	3	-3	-0.0016	-0.0351	-0.0785
-1.0700	-3.4226	0.0000	3	1	0.0200	0.0033	-0.0144
-1.7288	-0.9981	0.0000	3	1	0.0605	0.0576	-0.0942
-2.7068	1.5628	0.0000	3	1	0.2715	0.0245	-0.0295
-2.7525	1.1751	0.0000	3	3	0.1981	0.0427	0.0339
0.0000	2.3080	0.0000	3	3	0.3910	0.2861	0.1514
0.0000	1.9962	0.0000	3	1	0.0605	0.0576	-0.0942
2.3939	1.7962	0.0000	3	3	0.1981	0.0427	0.0339
-1.9690	1.1368	0.0000	3	3	0.2002	0.0560	0.0364
-2.3575	-1.3611	0.2195	3	-1	1.1680	-0.0711	-0.3330
-2.3575	-1.3611	-0.2195	3	-1	1.1680	-0.0711	-0.3330
0.0000	-1.5308	0.8679	3	-1	0.0157	-0.0030	-0.0090
0.0000	-1.5308	-0.8679	3	-1	0.0157	-0.0030	-0.0090
0.0000	2.9594	0.0000	3	3	0.4992	0.1480	0.1450
-2.9023	-1.6756	0.3170	3	-1	0.0069	-0.0128	-0.0742
-2.9023	-1.6756	-0.3170	3	-1	0.0069	-0.0128	-0.0742
-2.3558	-1.0788	0.0000	3	1	1.0170	0.1439	-0.2349
-1.9988	-1.1540	0.0000	3	3	0.3910	0.2861	0.1514
-2.3853	-0.4766	0.0000	3	-1	0.0011	-0.0384	-0.0827
2.3796	-0.5296	0.3153	3	-3	-0.0016	-0.0351	-0.0785
-0.3586	-2.9713	0.0000	3	3	0.1981	0.0427	0.0339
2.3796	-0.5296	-0.3153	3	-3	-0.0016	-0.0351	-0.0785
-2.6032	-1.9907	0.0000	3	1	0.0241	0.0120	-0.0863
-2.3939	1.7962	0.0000	3	3	0.1981	0.0427	0.0339
2.3994	1.3853	0.3639	3	-1	0.3315	-0.0476	-0.0509
-2.1122	-1.5008	0.0000	3	1	1.0170	0.1439	-0.2349
2.3994	1.3853	-0.3639	3	-1	0.3315	-0.0476	-0.0509
0.7799	2.3040	0.0000	3	-1	0.0011	-0.0384	-0.0827
-1.6484	-1.7960	0.3153	3	-3	-0.0016	-0.0351	-0.0785
-1.6484	-1.7960	-0.3153	3	-3	-0.0016	-0.0351	-0.0785
1.0700	-3.4226	0.0000	3	1	0.0200	0.0033	-0.0144
2.7898	1.8746	-1.6163	3	-1	0.0029	-0.0001	-0.0045
2.7898	1.8746	1.6163	3	-1	0.0029	-0.0001	-0.0045
0.0000	3.4183	0.0000	3	-3	-0.0051	-0.0204	-0.0655
0.0000	-3.3389	-1.6560	3	1	0.0026	0.0001	-0.0043
0.0000	-3.3389	1.6560	3	1	0.0026	0.0001	-0.0043
-0.7799	2.3040	0.0000	3	-1	0.0011	-0.0384	-0.0827
-2.3796	-0.5296	-0.3154	3	-3	-0.0016	-0.0351	-0.0785
-2.3796	-0.5296	0.3154	3	-3	-0.0016	-0.0351	-0.0785

2.8916	1.6695	-1.6560	3	1	0.0026	0.0001	-0.0043
2.8916	1.6695	1.6560	3	1	0.0026	0.0001	-0.0043
2.5629	-1.4797	0.0000	3	3	0.4992	0.1480	0.1450
2.2630	-1.3066	0.8748	3	-1	0.0007	-0.0144	-0.0751
2.2630	-1.3066	-0.8748	3	-1	0.0007	-0.0144	-0.0751
2.7525	1.1751	0.0000	3	3	0.1981	0.0427	0.0339
2.4291	2.6379	0.0000	3	1	0.0200	0.0033	-0.0144
0.7312	2.3256	-0.3153	3	-3	-0.0016	-0.0351	-0.0785
0.7312	2.3256	0.3153	3	-3	-0.0016	-0.0351	-0.0785
0.3586	-2.9713	0.0000	3	3	0.1981	0.0427	0.0339
-0.3397	-2.5051	0.0000	3	1	0.3133	0.0391	-0.0384
0.3397	-2.5051	0.0000	3	1	0.3133	0.0391	-0.0384
2.9603	-1.7091	0.0000	3	-3	-0.0051	-0.0204	-0.0655
0.0000	-2.2736	0.0000	3	3	0.2002	0.0560	0.0364
1.6484	-1.7960	0.3153	3	-3	-0.0016	-0.0351	-0.0785
1.6484	-1.7960	-0.3153	3	-3	-0.0016	-0.0351	-0.0785
1.6054	-1.8274	0.0000	3	-1	0.0011	-0.0384	-0.0827
0.0000	-3.1256	0.0000	3	1	0.2715	0.0245	-0.0295
-0.4224	3.2497	0.0000	3	1	0.0241	0.0120	-0.0863
1.9988	-1.1540	0.0000	3	3	0.3910	0.2861	0.1514
2.9023	-1.6756	0.3170	3	-1	0.0069	-0.0128	-0.0742
2.9023	-1.6756	-0.3170	3	-1	0.0069	-0.0128	-0.0742
2.3853	-0.4766	0.0000	3	-1	0.0011	-0.0384	-0.0827
1.9996	1.5467	0.0000	3	1	0.3133	0.0391	-0.0384
2.6032	-1.9907	0.0000	3	1	0.0241	0.0120	-0.0863
0.0000	-2.7706	0.3639	3	-1	0.3315	-0.0476	-0.0509
0.0000	-2.7706	-0.3639	3	-1	0.3315	-0.0476	-0.0509
3.0255	-1.2591	0.0000	3	1	0.0241	0.0120	-0.0863
0.0000	2.7222	0.2195	3	-1	1.1680	-0.0711	-0.3330
0.0000	2.7222	-0.2195	3	-1	1.1680	-0.0711	-0.3330
0.2436	2.5796	0.0000	3	1	1.0170	0.1439	-0.2349
2.1122	-1.5008	0.0000	3	1	1.0170	0.1439	-0.2349
1.9690	1.1368	0.0000	3	3	0.2002	0.0560	0.0364
-0.2436	2.5796	0.0000	3	1	1.0170	0.1439	-0.2349
2.7068	1.5628	0.0000	3	1	0.2715	0.0245	-0.0295
2.3575	-1.3611	0.2195	3	-1	1.1680	-0.0711	-0.3330
2.3575	-1.3611	-0.2195	3	-1	1.1680	-0.0711	-0.3330
2.3393	0.9583	0.0000	3	1	0.3133	0.0391	-0.0384
2.3558	-1.0788	0.0000	3	1	1.0170	0.1439	-0.2349
$\sum_j n_j = 129$						$\sum_j \mathrm{ind}_j n_j = 1$	

C_8H_8							
-0.0001	-0.0000	2.6615	3	3	0.0001	0.0001	0.0001
-0.0001	-0.0000	-2.6615	3	3	0.0001	0.0001	0.0001
-3.5317	-1.1677	0.0000	3	3	0.5041	0.2130	0.0904
-3.5317	1.1677	0.0000	3	3	0.5041	0.2130	0.0904
-2.8788	-1.1839	0.0000	3	3	0.4212	0.1880	0.1294
-2.8788	1.1839	0.0000	3	3	0.4212	0.1880	0.1294
-1.1839	-2.8788	0.0000	3	3	0.4212	0.1880	0.1294
-1.1839	2.8788	0.0000	3	3	0.4212	0.1880	0.1294
-1.1677	-3.5317	0.0000	3	3	0.5041	0.2130	0.0904
-1.1677	3.5317	0.0000	3	3	0.5041	0.2130	0.0904
1.1839	-2.8788	0.0000	3	3	0.4212	0.1880	0.1294
1.1839	2.8788	0.0000	3	3	0.4212	0.1880	0.1294
1.1677	-3.5317	0.0000	3	3	0.5041	0.2130	0.0904
1.1677	3.5317	0.0000	3	3	0.5041	0.2130	0.0904
2.8788	-1.1839	0.0000	3	3	0.4212	0.1880	0.1294

2.8788	1.1839	0.0000	3	3	0.4212	0.1880	0.1294
3.5317	-1.1677	0.0000	3	3	0.5041	0.2130	0.0904
3.5317	1.1677	0.0000	3	3	0.5041	0.2130	0.0904
-3.4243	2.2544	0.0000	3	1	0.0293	0.0075	-0.0399
-3.4243	-2.2544	0.0000	3	1	0.0293	0.0075	-0.0399
-4.1560	-0.8860	0.0000	3	1	0.0290	0.0109	-0.0418
-4.1560	0.8860	0.0000	3	1	0.0290	0.0109	-0.0418
-0.0000	0.0000	0.0000	3	1	0.0009	0.0009	-0.0001
-2.2544	-3.4243	0.0000	3	1	0.0293	0.0075	-0.0399
-2.2544	3.4243	0.0000	3	1	0.0293	0.0075	-0.0399
-2.2654	-1.0574	0.0000	3	1	0.0353	0.0160	-0.0401
-2.2654	1.0574	0.0000	3	1	0.0353	0.0160	-0.0401
-3.2338	-0.9666	0.0000	3	1	0.8796	0.2659	-0.1187
-3.2338	0.9666	0.0000	3	1	0.8796	0.2659	-0.1187
2.2654	-1.0574	0.0000	3	1	0.0353	0.0160	-0.0401
-1.0574	2.2654	0.0000	3	1	0.0353	0.0160	-0.0401
2.2654	1.0574	0.0000	3	1	0.0353	0.0160	-0.0401
-1.0574	-2.2654	0.0000	3	1	0.0353	0.0160	-0.0401
1.0574	2.2654	0.0000	3	1	0.0353	0.0160	-0.0401
1.0574	-2.2654	0.0000	3	1	0.0353	0.0160	-0.0401
-0.8860	-4.1560	0.0000	3	1	0.0290	0.0109	-0.0418
-0.8860	4.1560	0.0000	3	1	0.0290	0.0109	-0.0418
0.8860	-4.1560	0.0000	3	1	0.0290	0.0109	-0.0418
-0.9666	-3.2338	0.0000	3	1	0.8796	0.2659	-0.1187
-0.9666	3.2338	0.0000	3	1	0.8796	0.2659	-0.1187
0.8860	4.1560	0.0000	3	1	0.0290	0.0109	-0.0418
2.2544	-3.4243	0.0000	3	1	0.0293	0.0075	-0.0399
2.2544	3.4243	0.0000	3	1	0.0293	0.0075	-0.0399
0.9666	-3.2338	0.0000	3	1	0.8796	0.2659	-0.1187
0.9666	3.2338	0.0000	3	1	0.8796	0.2659	-0.1187
3.4243	-2.2544	0.0000	3	1	0.0293	0.0075	-0.0399
3.4243	2.2544	0.0000	3	1	0.0293	0.0075	-0.0399
3.2338	-0.9666	0.0000	3	1	0.8796	0.2659	-0.1187
3.2338	0.9666	0.0000	3	1	0.8796	0.2659	-0.1187
4.1560	-0.8860	0.0000	3	1	0.0290	0.0109	-0.0418
4.1560	0.8860	0.0000	3	1	0.0290	0.0109	-0.0418
-2.3793	3.1715	-0.5648	3	-1	0.0129	-0.0131 $\pm i$	0.0049
-2.3793	3.1715	0.5648	3	-1	0.0129	-0.0131 $\pm i$	0.0049
-2.3793	-3.1715	-0.5648	3	-1	0.0129	-0.0131 $\pm i$	0.0049
-2.3793	-3.1715	0.5648	3	-1	0.0129	-0.0131 $\pm i$	0.0049
-4.1160	-0.7493	0.6693	3	-1	0.0114	-0.0157 $\pm i$	0.0055
-4.1160	-0.7493	-0.6693	3	-1	0.0114	-0.0157 $\pm i$	0.0055
-4.1160	0.7493	-0.6693	3	-1	0.0114	-0.0157 $\pm i$	0.0055
-4.1160	0.7493	0.6693	3	-1	0.0114	-0.0157 $\pm i$	0.0055
-3.2248	-0.0000	-3.0501	3	-1	0.0097	-0.0066	-0.0251
-3.0501	-1.7610	-0.8411	3	-1	0.0097	-0.0066	-0.0251
-3.0501	1.7610	0.8411	3	-1	0.0097	-0.0066	-0.0251
-3.0501	1.7610	-0.8411	3	-1	0.0097	-0.0066	-0.0251
0.0000	-1.7655	-0.9516	3	-1	0.0093	-0.0115	-0.0175
0.0000	-1.7655	0.9516	3	-1	0.0093	-0.0115	-0.0175
0.0000	1.7655	-0.9516	3	-1	0.0093	-0.0115	-0.0175
0.0000	1.7655	0.9516	3	-1	0.0093	-0.0115	-0.0175
1.5290	-0.8827	0.9516	3	-1	0.0093	-0.0115	-0.0175
1.5290	-0.8827	-0.9516	3	-1	0.0093	-0.0115	-0.0175
1.5290	0.8827	0.9516	3	-1	0.0093	-0.0115	-0.0175
1.5290	0.8827	-0.9516	3	-1	0.0093	-0.0115	-0.0175
3.0501	1.7610	-0.8411	3	-1	0.0097	-0.0066	-0.0251

3.0501	1.7610	0.8411	3	-1	0.0097	-0.0066	-0.0251
3.0501	-1.7610	-0.8411	3	-1	0.0097	-0.0066	-0.0251
3.0501	-1.7610	0.8411	3	-1	0.0097	-0.0066	-0.0251
-2.3905	-1.3802	0.2097	3	-1	0.8930	-0.1159	-0.2191
-2.3905	-1.3802	-0.2097	3	-1	0.8930	-0.1159	-0.2191
-2.3905	1.3802	0.2097	3	-1	0.8930	-0.1159	-0.2191
-2.3905	1.3802	-0.2097	3	-1	0.8930	-0.1159	-0.2191
0.0000	-2.7603	-0.2097	3	-1	0.8930	-0.1159	-0.2191
0.0000	-2.7603	0.2097	3	-1	0.8930	-0.1159	-0.2191
0.0000	2.7603	-0.2097	3	-1	0.8930	-0.1159	-0.2191
0.0000	2.7603	0.2097	3	-1	0.8930	-0.1159	-0.2191
2.3905	-1.3802	-0.2097	3	-1	0.8930	-0.1159	-0.2191
2.3905	-1.3802	0.2097	3	-1	0.8930	-0.1159	-0.2191
2.3905	1.3802	-0.2097	3	-1	0.8930	-0.1159	-0.2191
2.3905	1.3802	0.2097	3	-1	0.8930	-0.1159	-0.2191
0.0000	2.6508	0.0000	3	-3	-6.9640	-6.9880	-7.0630
2.2957	1.3254	0.0000	3	-3	-6.9640	-6.9880	-7.0630
-2.2957	1.3254	0.0000	3	-3	-6.9640	-6.9880	-7.0630
2.2957	-1.3254	0.0000	3	-3	-6.9640	-6.9880	-7.0630
3.4474	1.9904	0.0000	3	-3	-0.0163	-0.0190	-0.0295
0.0000	3.9808	0.0000	3	-3	-0.0163	-0.0190	-0.0295
-2.2957	-1.3254	0.0000	3	-3	-6.9640	-6.9880	-7.0630
-3.4474	1.9904	0.0000	3	-3	-0.0163	-0.0190	-0.0295
0.0000	-2.6508	0.0000	3	-3	-6.9640	-6.9880	-7.0630
3.4474	-1.9904	0.0000	3	-3	-0.0163	-0.0190	-0.0295
-3.4474	-1.9904	0.0000	3	-3	-0.0163	-0.0190	-0.0295
0.0000	-3.9808	0.0000	3	-3	-0.0163	-0.0190	-0.0295
-2.3184	0.0000	0.0000	3	-3	-0.0009	-0.0348	-0.0497
2.3184	0.0000	0.0000	3	-3	-0.0009	-0.0348	-0.0497
-1.1592	-2.0078	0.0000	3	-3	-0.0009	-0.0348	-0.0497
-1.1592	2.0078	0.0000	3	-3	-0.0009	-0.0348	-0.0497
1.1592	2.0078	0.0000	3	-3	-0.0009	-0.0348	-0.0497
1.1592	-2.0078	0.0000	3	-3	-0.0009	-0.0348	-0.0497
$\sum_j n_j = 137$						$\sum_j \text{ind}_j n_j = 5$	

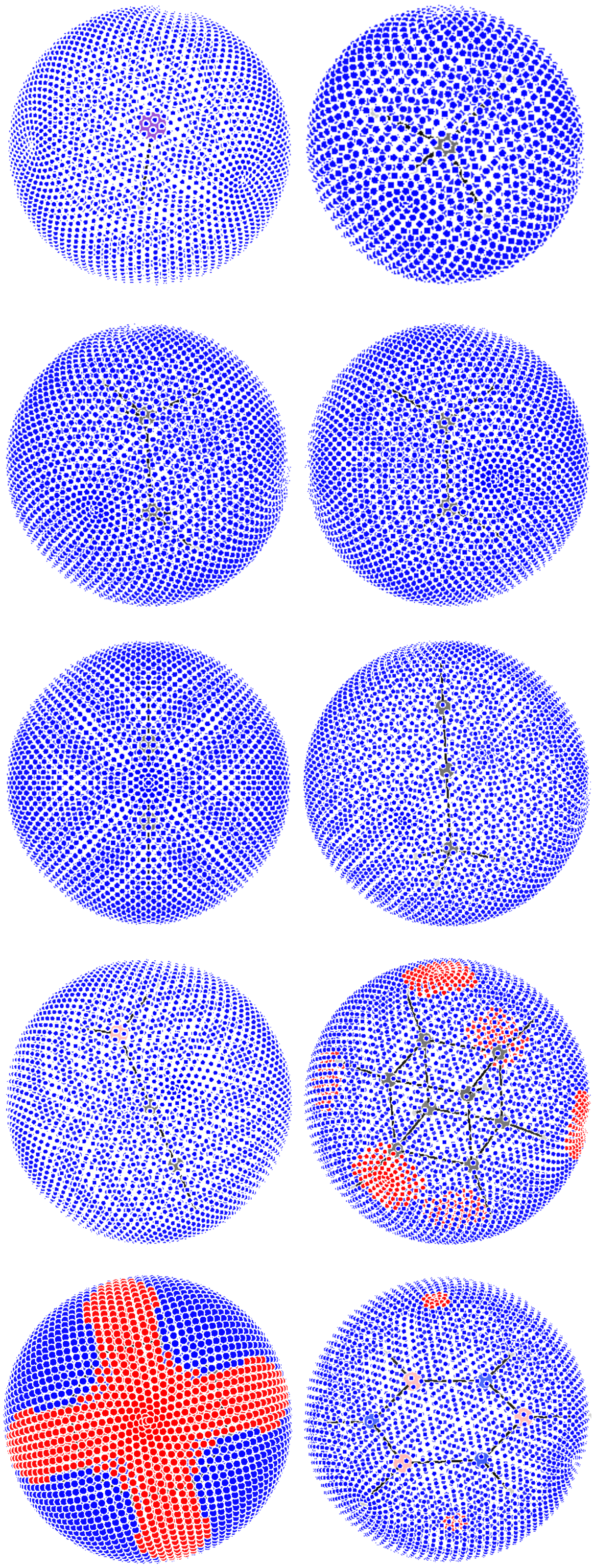

Figure S2. Spheres of 5 au radius containing all isolated CPs of 10 molecules studied in the article. Points on the surface have been coloured in red/blue according to whether the flux of the force, $\langle \boldsymbol{F} \rangle \cdot dS$ is outward/inward oriented. From left to right, top to bottom: LiH, CH_4, C_2H_6, C_2H_4, C_2H_2, C_3H_5, BC_2H_4, C_8H_8 (cubane), C_8H_8 (planarized cyclooctatetraene), $B_3N_3H_6$.
